# Strain-Dependent Lactic Fermentation Primes Milk Proteins for Gastrointestinal Digestion Under Age-Specific Physiological Conditions

**DOI:** 10.3390/foods15203712

**Published:** 2026-10-19

**Authors:** Rafaela Ansiliero, Leandra Oliveira Xavier Albiero, Aniela Pinto Kempka

**Affiliations:** 1 Multicenter Program in Postgraduate in Biochemistry and Molecular Biology, College of Agronomy and Veterinary, Santa Catarina State University, Lages 88520-000, SC, Brazil; rafaela.a@edu.udesc.br (R.A.); leandraoliveiraxa@outlook.com (L.O.X.A.); 2 Department of Food Engineering and Chemical Engineering, Santa Catarina State University, Pinhalzinho 89870-000, SC, Brazil

**Keywords:** *Lactobacillus helveticus*, *Lactobacillus delbrueckii* subsp. *bulgaricus*, β-lactoglobulin, casein, immunoreactivity, digestive maturity, infant and adult digestion models

## Abstract

Lactic fermentation modifies milk proteins through the proteolytic activity of lactic acid bacteria, potentially altering their susceptibility to gastrointestinal digestion. This study evaluated how fermentation by *Lactobacillus delbrueckii* and *Lactobacillus helveticus*, individually and in co-culture, affects the structure of bovine milk proteins, their subsequent gastrointestinal digestion, and residual immunoreactivity. Milk was fermented with *L. delbrueckii*, *L. helveticus*, or their co-culture. Residual immunoreactivity of caseins (CNs) and β-lactoglobulin (βLG) was determined by ELISA, and protein profiles were evaluated by SDS-PAGE before and after in vitro gastrointestinal digestion using age-specific physiological models representative of 1-, 3-, and 6-month-old infants and adults. Fermentation with *L. delbrueckii* and the co-culture reduced the ELISA-detectable CN fraction by 7.42% and 8.85%, respectively, while *L. delbrueckii* was the only treatment to significantly decrease residual βLG immunoreactivity (2.17%). The effects of fermentation were most evident in the infant digestion models. Fermentation by *L. delbrueckii* was associated with more pronounced reductions in bands within the molecular weight regions expected for caseins and whey proteins during gastric digestion in the 1- and 3-month-old infant models, whereas these differences progressively diminished with digestive maturity. Throughout all digestion models, bands within the molecular weight range expected for βLG remained among the most persistent protein bands during gastric digestion. In silico analyses predicted preferential hydrolysis of caseins, the release of peptides with potential biological activities, and the differential persistence of linear allergenic epitope sequences among milk proteins. Strain-dependent lactic fermentation partially remodeled milk proteins before digestion, influencing their subsequent gastrointestinal behavior. The results indicate that the effects of fermentation depend not only on strain-specific protein modifications but also on the physiological conditions under which digestion occurs, providing new insights into the interaction between fermentation-induced protein remodeling and digestive maturity.

## 1. Introduction

Cow’s milk proteins are recognized for their high nutritional and functional value and are major ingredients of infant formulas used when breastfeeding is not possible or insufficient [1,2,3,4,5]. The structural organization of these proteins strongly influences their susceptibility to gastrointestinal digestion, making the relationship between protein structure and digestive behavior a key consideration in the development of biotechnological strategies aimed at modulating their nutritional and biological properties.

Bovine milk proteins comprise two major fractions: caseins (CNs), including β-casein (β-CN), αS1-casein (αS1-CN), αS2-casein (αS2-CN), and κ-casein (κ-CN), and whey proteins, of which β-lactoglobulin (βLG) and α-lactalbumin (αLA) are the most abundant [1,6,7]. Among the major milk proteins, βLG exhibits high resistance to gastrointestinal digestion because of its compact globular structure, predominantly composed of β-sheets and stabilized by intramolecular disulfide bonds [8,9,10,11]. This resistance is more pronounced in infants owing to their higher gastric pH and lower pepsin and pancreatic enzyme activities than in adults [12,13]. Consequently, βLG may persist longer during gastrointestinal transit, increasing the exposure of allergenic epitopes to the immune system. This characteristic is particularly relevant because βLG is one of the major allergenic proteins in bovine milk and is not naturally present in human milk [1,8,14,15,16,17].

Lactic fermentation is a well-established biotechnological strategy for modifying milk proteins through the proteolytic activity of lactic acid bacteria (LAB) [14,18,19]. Among LAB, *L. delbrueckii* and *L. helveticus* possess proteolytic systems capable of hydrolyzing high-molecular-weight proteins into peptides and amino acids [20,21]. This activity promotes peptide bond cleavage and structural remodeling of milk proteins, potentially modifying their behavior during gastrointestinal digestion [18]. Fermentation may therefore contribute to the degradation of allergenic epitopes [22] and the release of bioactive peptides with diverse physiological properties, increasing the functional potential of fermented dairy products [23,24]. These peptides have been associated with antioxidant, anti-inflammatory, immunomodulatory, antimicrobial, antiviral, neuroprotective, anticancer, and intestinal barrier-protective activities [25,26,27,28]. Because fermentation precedes gastrointestinal digestion, the structural modifications introduced by microbial proteolysis may influence the subsequent accessibility of digestive enzymes to milk proteins, particularly across different physiological conditions [29].

Although the proteolytic potential of lactic acid bacteria has been extensively investigated, most studies have focused on protein hydrolysis during fermentation or the release of bioactive peptides [30]. Whether the structural modifications induced during fermentation persist and influence the subsequent gastrointestinal digestion of milk proteins under physiologically relevant conditions representing different stages of digestive maturity remains poorly understood. This question is particularly relevant during early life, when the limited digestive capacity of infants may amplify or attenuate the effects of fermentation-induced protein modifications during gastrointestinal digestion [30,31].

Whether strain-dependent lactic fermentation influences the subsequent gastrointestinal digestion of milk proteins under physiologically relevant conditions representative of different stages of digestive maturity remains unclear. We hypothesized that fermentation by different *Lactobacillus* strains, individually and in co-culture, partially remodels milk proteins before digestion, thereby altering their susceptibility to gastrointestinal proteolysis and residual immunoreactivity. Accordingly, this study aimed to evaluate how fermentation by *L. delbrueckii* and *L. helveticus*, individually and in co-culture, affects the structural characteristics of bovine milk proteins, their subsequent in vitro gastrointestinal digestion under physiological conditions representative of 1-, 3-, and 6-month-old infants and adults, and their residual immunoreactivity.

## 2. Materials and Methods

### 2.1. Materials and Chemicals

Lyophilized *L. delbrueckii* subsp. *bulgaricus* CCT8106 and *L. helveticus* LH76 cultures were obtained from a compounding pharmacy in Chapecó, Santa Catarina, Brazil, with an approximate concentration of 1 × 10^9^ bacterial cells per capsule. The selection of the microorganisms was based on a literature search indicating that lactic fermentation with these strains has the potential to reduce the immunoreactivity, antigenicity, and allergenicity of cow’s milk proteins [16,32,33,34]. Cultures were stored in Tryptic Soy Broth (TSB; Oxoid Ltd., Basingstoke, UK) supplemented with 25% (*v*/*v*) glycerol at −83 °C and on Tryptic Soy Agar (TSA; Oxoid Ltd., Basingstoke, UK) slants at 4 °C. De Man–Rogosa–Sharpe (MRS) broth and agar (Merck, Darmstadt, Germany) were used for culture reactivation and propagation. Instant skim milk powder (Elegê, Teutônia, RS, Brazil; lot 16/09A2; 3.4% protein (*w*/*v*), 5.0% carbohydrates (*w*/*v*), 0.05% sodium (*w*/*v*), and 0.112% calcium (*w*/*v*)) was used as the fermentation substrate and reconstituted as described below. This matrix was selected to provide a standardized processed dairy system, ensuring that the non-fermented control and all fermentation treatments shared the same processing history and initial protein matrix. In vitro gastrointestinal digestion was performed using pepsin (ACS Científica^®^, Sumaré, SP, Brazil), pancreatin, and bile salts (Sigma-Aldrich^®^, Cotia, SP, Brazil). Residual βLG and CN immunoreactivity were determined using the RIDASCREEN^®^ β-Lactoglobulin (Art. No. R4901) and RIDASCREEN^®^ FAST Casein (Art. No. R4612) ELISA kits (R-Biopharm AG, Darmstadt, Germany), respectively. Analytical-grade hydrochloric acid, calcium chloride, and sodium chloride were purchased from Biotec^®^ (São José dos Pinhais, PR, Brazil); sodium bicarbonate and sodium hydroxide from Dinâmica^®^ (Indaiatuba, SP, Brazil); and glycerol from Synth^®^ (São Paulo, SP, Brazil). SDS-PAGE analyses were performed using glycine, bromophenol blue, 30% bis-acrylamide, β-mercaptoethanol, *N,N,N′,N′*-tetramethylethylenediamine (TEMED), ammonium persulfate (APS), and sodium dodecyl sulfate (SDS) (Sigma-Aldrich^®^, Cotia, SP, Brazil); Tris(hydroxymethyl)aminomethane (Tris; Neon^®^, Suzano, SP, Brazil); Fast Blue (Scienco Biotech^®^, Lages, SC, Brazil); and an unstained protein molecular weight marker (14.4–116 kDa; Thermo Scientific, Art. No. 26610). This study was registered in the Brazilian National System for the Management of Genetic Heritage and Associated Traditional Knowledge (SisGen; registration code A3D4857) in accordance with Brazilian Law No. 13,123/2015 and Decree No. 8,772/2016.

### 2.2. Molecular Identification of Bacterial Strains

Lyophilized bacterial cultures obtained from the compounding pharmacy were cultivated on TSA slants at 37 °C for 24 h and subsequently submitted for molecular identification based on 16S rRNA gene sequencing at Desenvolvimento Sustentável e Monitoramento Ambiental (DMSA, Mogi das Cruzes, SP, Brazil). Genomic DNA was extracted using a commercial kit, followed by amplification of the 16S rRNA gene by polymerase chain reaction (PCR) and Sanger sequencing using an Applied Biosystems 3730XL DNA Analyzer [35,36]. A phylogenetic tree was constructed from the 16S rRNA gene sequences using MEGA version 11 [37]. Evolutionary relationships were inferred using the Neighbor-Joining method with the Jukes–Cantor substitution model, and branch support was assessed by bootstrap analysis with 1000 replicates.

### 2.3. Activation of Bacterial Strains and Growth Profile

The bacterial strains were cultured in 10 mL of MRS broth at 37 °C for 24 h under static conditions (SL 200/334, Solab, Piracicaba, SP, Brazil). The resulting cultures were standardized to 0.5 McFarland turbidity (approximately 8 log CFU/mL) using sterile 0.85% (*w*/*v*) saline solution and stored at 10 °C for up to 24 h before use. Bacterial growth profiles were determined in triplicate by inoculating MRS broth with 1% (*v*/*v*) of the standardized cultures. Cell growth was monitored by measuring the optical density at 600 nm (OD600) every 2 h during an 18 h incubation period using a Varioskan™ LUX microplate reader (Thermo Fisher Scientific, Waltham, MA, USA) [27]. The resulting growth curves were used to define the fermentation time adopted in the subsequent experiments. Based on these results, an incubation period of 14 h was selected for all fermentation assays (Figure S1, Supplementary Materials).

### 2.4. Preparation of Reconstituted Milk and Fermentation Process

Instant skim milk powder (Elegê, lot 16/09A2) was reconstituted in Type I ultrapure water to a final concentration of 10% (*w*/*v*) under aseptic conditions at room temperature (20–25 °C). After complete dissolution of the milk powder, the initial pH was measured using a calibrated pH meter (MY140, Mylabor Equipamentos, Tatuapé, SP, Brazil; pH 6.47). To minimize microbial interference during the fermentation assays, the reconstituted milk was autoclaved at 105 °C for 15 min [38], cooled to room temperature, and the pH was measured again (pH 6.70). Fermentation was carried out by inoculating the reconstituted milk with standardized cultures (approximately 8 log CFU/mL) of *L. delbrueckii* (LD) or *L. helveticus* (LH) at a final inoculum of 1% (*v*/*v*). For co-culture fermentation (LD + LH), each strain was inoculated at 0.5% (*v*/*v*), resulting in the same final inoculum (1% *v*/*v*). Samples were incubated at 37 °C for 14 h under static conditions. Four experimental groups were evaluated: a non-fermented control and three fermentation treatments (LD, LH, and LD + LH), each prepared as independent biological replicates (*n* = 3). Fermentation progress was monitored by measuring pH, titratable acidity, and LAB counts in fermented and reconstituted milk samples at 0, 4, 8, 12, and 14 h. The pH was measured in triplicate using a calibrated pH meter (MY140, Mylabor Equipamentos, Tatuapé, SP, Brazil). The percentage reduction in pH was calculated as the difference between the initial and final values divided by the initial value and multiplied by 100.

Titratable acidity was determined in triplicate according to Shahein et al. [39]. A 10 g aliquot of each sample was homogenized with 10 mL of distilled water and titrated with 0.1 N NaOH using phenolphthalein as the indicator until a persistent pale pink endpoint was reached. Results are expressed as a percentage of lactic acid and calculated according to Equation (1), where *V* is the volume of NaOH consumed during titration (mL), *N* is the normality of the NaOH solution, *f* is the conversion factor, and *m* is the sample mass (g).

(1) $$\mathrm{Titratable}\;\mathrm{acidity}\;\left(\%\;\mathrm{lactic}\;\mathrm{acid}\right)=\left(\frac{V\times N\times f}{m}\right)\times 100$$

LAB enumeration was performed in duplicate according to Ansiliero et al. [40], with minor modifications. A 10 g aliquot of each sample was homogenized with 90 mL of sterile 0.85% (*w*/*v*) saline solution to obtain a 10^−1^ dilution. Serial decimal dilutions were prepared in the same diluent, and 100 μL aliquots of the appropriate dilutions were spread onto MRS agar plates, followed by incubation at 37 °C for 48 h. Results are expressed as log CFU/mL.

### 2.5. Determination of Soluble Protein Concentration

Soluble protein concentration was determined in triplicate using the Bradford assay [41]. Samples were centrifuged at 10,000 rpm for 10 min at 4 °C (SL-703, Solab, Piracicaba, SP, Brazil; approximately 6700× *g*, rotor radius 6 cm), and the supernatants were collected for analysis. When necessary, samples were diluted with ultrapure water to ensure that absorbance values remained within the linear range of the assay. Aliquots (10 μL) of samples or bovine serum albumin (BSA) standards were transferred to 96-well microplates and mixed with 200 μL of Bradford reagent. After incubation for 5 min at room temperature (22–25 °C), absorbance was measured at 595 nm using a Varioskan™ LUX microplate reader (Thermo Fisher Scientific, Waltham, MA, USA). A standard curve was prepared using BSA solutions ranging from 0.15 to 0.75 mg/mL (six concentration points) and analyzed under the same experimental conditions as the samples. Soluble protein concentrations were calculated from the corresponding standard curve.

### 2.6. Sodium Dodecyl Sulfate–Polyacrylamide Gel Electrophoresis (SDS-PAGE)

Protein profiles of reconstituted and fermented milk samples were analyzed by sodium dodecyl sulfate–polyacrylamide gel electrophoresis (SDS-PAGE) [42]. Electrophoresis was performed using a Vert-i10 system (Loccus Biotecnologia, Cotia, SP, Brazil) with 4% stacking and 12% resolving gels prepared from a 30% bis-acrylamide solution. For each treatment, equal aliquots from the independent biological replicates were pooled to obtain a representative sample for electrophoretic analysis. Samples were mixed 1:1 (*v*/*v*) with 2× Laemmli sample buffer (125 mM Tris-HCl, pH 6.8, 4% SDS, 20% glycerol, 0.01% bromophenol blue, and 10% β-mercaptoethanol), denatured at 95 °C for 10 min, and immediately cooled on ice. Aliquots (10 μL) were loaded onto the gels together with an unstained protein molecular weight marker (14.4–116 kDa; Thermo Scientific, Art. No. 26610). Electrophoretic separation was performed in Tris–glycine–SDS running buffer (25 mM Tris, 192 mM glycine, and 0.1% SDS) at a constant voltage of 180 V for approximately 60 min using an LPS-300V power supply (Loccus Biotecnologia, Cotia, SP, Brazil). Following electrophoresis, the gels were rinsed with distilled water and stained overnight under gentle agitation with a Fast Blue staining solution containing 0.125 g of enhancer per 10 mL of staining solution. The gels were subsequently destained by successive washes with distilled water until a clear background was obtained. The SDS-PAGE profiles were interpreted qualitatively based on the presence, absence, and visual intensity of the observed bands.

### 2.7. Determination of Residual Immunoreactivity of CN and βLG by ELISA

Residual immunoreactivity of CN and βLG was evaluated using the RIDASCREEN^®^ FAST Casein (Art. No. R4612) and RIDASCREEN^®^ β-Lactoglobulin (Art. No. R4901) commercial ELISA kits (R-Biopharm AG, Darmstadt, Germany), respectively. The RIDASCREEN^®^ FAST Casein assay is a sandwich ELISA designed to detect antibody-recognized casein, whereas the RIDASCREEN^®^ β-Lactoglobulin assay is a competitive ELISA developed to quantify immunoreactive βLG, including native, processed, and hydrolyzed forms, as well as antibody-recognized peptide fragments. Both assays were used to evaluate the residual immunoreactivity of milk proteins following lactic fermentation. All assays were performed according to the manufacturer’s instructions, and all samples were analyzed in biological duplicate.

### 2.8. In Vitro Gastrointestinal Digestion

**Figure 1:**
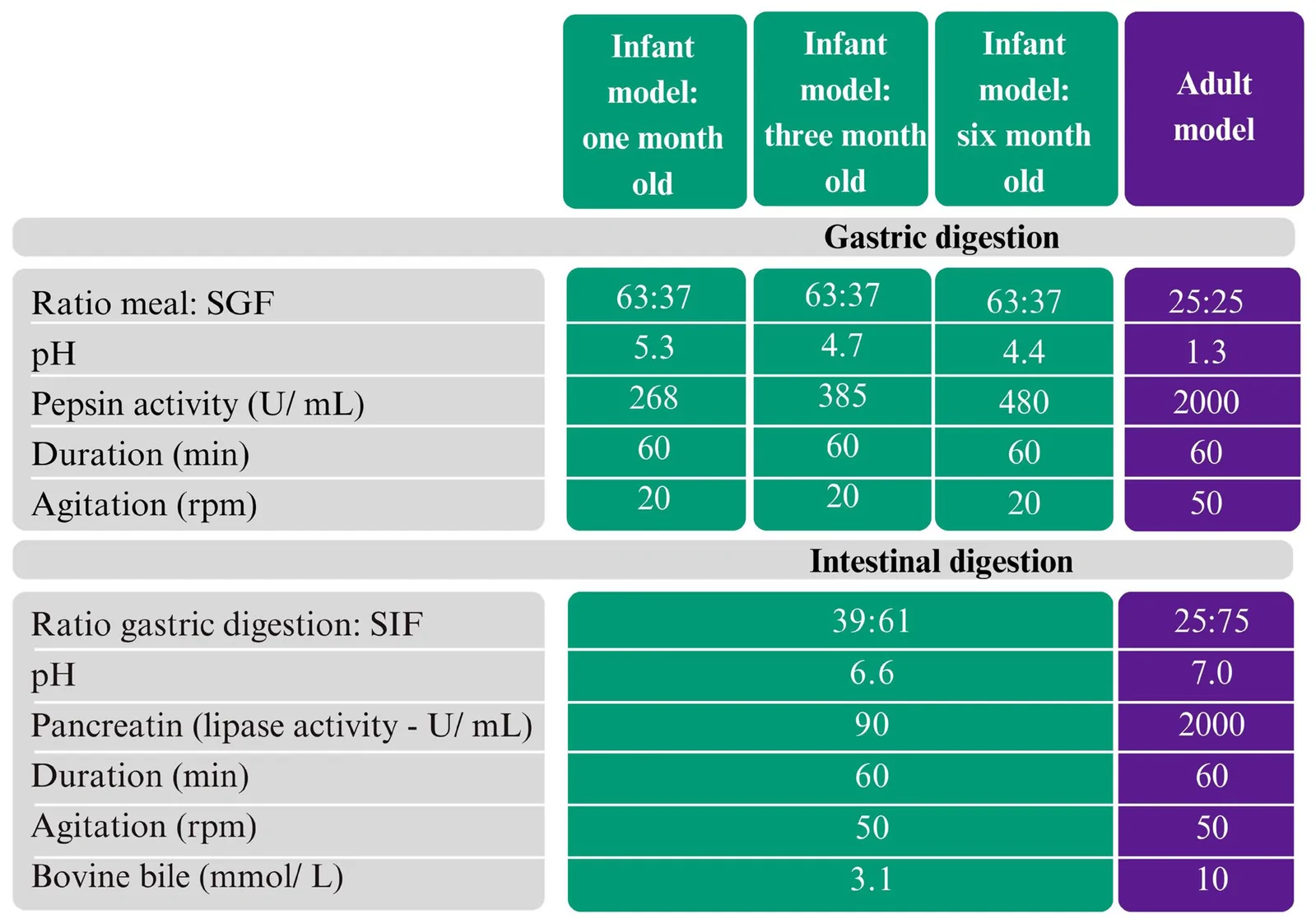
Experimental design and physiological conditions adopted for the in vitro gastrointestinal digestion models representative of 1-, 3-, and 6-month-old infants and adults. Gastric and intestinal phases were performed sequentially under age-specific conditions. Note: SGF, simulated gastric fluid; SIF, simulated intestinal fluid.

The effects of lactic fermentation on milk protein digestibility were evaluated using in vitro gastrointestinal digestion models representative of 1-, 3-, and 6-month-old infants and adults, adapted from the protocols described by Miltenburg et al. [12] and Ménard et al. [43]. The experimental design and physiological conditions adopted for each digestion model are summarized in Figure 1. Digestion experiments were performed in biological duplicate and consisted of sequential gastric and intestinal phases. The oral phase was omitted because liquid foods have a short residence time in the oral cavity, resulting in negligible oral proteolysis [44]. At the end of each digestion phase, aliquots were collected and immediately frozen at −83 °C to terminate enzymatic activity. Samples were subsequently freeze-dried (−60 °C, 0.05 mTorr, 24 h; TFD5503, Ilshin Lab Co. Ltd., Yangju, Republic of Korea) and stored at −83 °C until SDS-PAGE and ELISA analyses.

#### 2.8.1. Infant Gastric Digestion Models (1, 3, and 6 Months)

The gastric digestion models representative of 1-, 3-, and 6-month-old infants were established according to Miltenburg et al. [12]. Simulated gastric fluid (SGF), consisting of 94 mM NaCl, was mixed with the samples at a meal-to-SGF ratio of 63:37 (*v*/*v*). Gastric digestion was performed at pH 5.3 with a final pepsin activity of 268 U/mL for the 1-month-old model, at pH 4.7 with 385 U/mL for the 3-month-old model, and at pH 4.4 with 480 U/mL for the 6-month-old model. Gastric lipase was not included because skim milk powder, which contains negligible lipid content, was used as the substrate.

#### 2.8.2. Adult Gastric Digestion Model

The adult gastric digestion model was established according to the protocol described by Ménard et al. [43]. SGF, consisting of 94 mM NaCl, was mixed with the samples at a meal-to-SGF ratio of 50:50 (*v*/*v*). Gastric digestion was performed at pH 1.3 with a final pepsin activity of 2000 U/mL. As in the infant models, gastric lipase was not included because skim milk powder, which contains negligible lipid content, was used as the substrate.

#### 2.8.3. In Vitro Gastric Digestion Procedure

Milk samples were mixed with the corresponding SGF, and the pH was adjusted with 1 M HCl to the target value established for each digestion model (Section 2.8.1 and Section 2.8.2). Gastric digestion was initiated by the addition of pepsin and carried out at 37 °C for 60 min under continuous agitation (20 rpm for the infant models and 50 rpm for the adult model). At the end of the gastric phase, enzymatic activity was immediately terminated by placing the reaction mixtures in an ice bath for 10 min.

#### 2.8.4. Infant Intestinal Digestion Model

The intestinal digestion models representative of 1-, 3-, and 6-month-old infants were established according to Ménard et al. [43]. SIF, consisting of 164 mM NaCl, 85 mM NaHCO_3_, and 3 mM CaCl_2_, was adjusted to pH 6.6. Following the gastric phase, the chyme was neutralized to pH 6.6 with 1 M NaOH and mixed with SIF at a chyme-to-SIF ratio of 39:61 (*v*/*v*). Bovine bile extract and pancreatin were then added to obtain final concentrations of 3.1 mmol/L bile salts and a lipase activity of 90 U/mL in the digestion mixture.

#### 2.8.5. Adult Intestinal Digestion Model

The adult intestinal digestion model was established according to Ménard et al. [43]. Following the gastric phase, the chyme was neutralized to pH 7.0 with 1 M NaOH and mixed with SIF, adjusted to pH 7.0, at a chyme-to-SIF ratio of 25:75 (*v*/*v*). Bovine bile extract and pancreatin were then added to obtain final concentrations of 10 mmol/L bile salts and a lipase activity of 2000 U/mL in the digestion mixture.

#### 2.8.6. In Vitro Intestinal Digestion Procedure

Following completion of the gastric phase, the chyme was mixed with the corresponding SIF, after which bovine bile extract and pancreatin were added to initiate intestinal digestion. The reaction mixtures were incubated at 37 °C under continuous agitation (50 rpm) for 60 min. At the end of the intestinal phase, enzymatic activity was immediately terminated by placing the reaction mixtures in an ice bath for 10 min.

### 2.9. In Silico Simulation of Milk Protein Hydrolysis

The amino acid sequences of β-casein (β-CN), αS1-casein (αS1-CN), αS2-casein (αS2-CN), β-lactoglobulin (βLG), α-lactalbumin (αLA), and bovine serum albumin (BSA) were retrieved from the BIOPEP-UWM database, which also provides experimentally described allergenic epitopes for these proteins (Table S1, Supplementary Materials) [45]. In silico hydrolysis was performed using the enzyme action module available in BIOPEP-UWM. Lactocepin (BRENDA EC 3.4.21.96), a cell envelope-associated serine endopeptidase, was selected as the hydrolytic model because its substrate specificity is consistent with that reported for the major cell-envelope proteinases (CEPs) of *L. helveticus* and *L. delbrueckii*, according to the BRENDA enzyme database [46]. The simulation provided theoretical estimates of the degree of hydrolysis, released peptides and amino acids, remaining potential linear allergenic epitope sequences, their positions within the protein sequences, and the predicted bioactive potential of the generated peptides according to the BIOPEP-UWM annotation criteria. The resulting datasets were exported to Microsoft Excel 365 (Microsoft Corp., Redmond, WA, USA) for organization, processing, and tabulation.

### 2.10. Statistical Analysis

Statistical analyses were performed using PAST software (version 4.3, Oslo, Norway) [47]. Data normality was assessed using the Shapiro–Wilk test. Normally distributed data (*p* ≥ 0.05) were analyzed using Student’s *t*-test or one-way analysis of variance (ANOVA), followed by Tukey’s multiple-comparison test when appropriate. Non-normally distributed data were analyzed using the Kruskal–Wallis test followed by Dunn’s multiple-comparison test. Statistical significance was established at *p* < 0.05. No formal a priori statistical power analysis was performed; therefore, statistical inferences were interpreted within the limits of the experimental sample sizes described for each analysis.

## 3. Results and Discussion

### 3.1. Molecular Identification of the Bacterial Strains

Based on 16S rRNA gene sequencing and phylogenetic clustering (Figure S2, Supplementary Materials), the microorganisms were identified as *L. delbrueckii* and *L. helveticus*, showing 99.20% and 99.29% sequence similarity with reference strains deposited in the NCBI GenBank database, respectively (Table 1). Both microorganisms presented 100% query coverage, supporting reliable taxonomic identification. Species of the genus *Lactobacillus* are Gram-positive, non-spore-forming bacteria widely used as starter and adjunct cultures in dairy fermentations because of their recognized safety status (GRAS) and well-established proteolytic systems, which contribute to the transformation of milk proteins during fermentation [48,49,50,51]. Although *L. delbrueckii* and *L. helveticus* belong to the same genus, they possess distinct cell-envelope proteinase (CEP) systems and intracellular peptidase repertoires, resulting in differences in substrate specificity and peptide release during milk fermentation [52]. These differences are particularly relevant in the present study because the extent and specificity of protein hydrolysis during fermentation may influence the structural characteristics of milk proteins and their subsequent susceptibility to gastrointestinal digestion.

### 3.2. Fermentation Performance and Protein Modification During Lactic Fermentation

**Figure 2:**
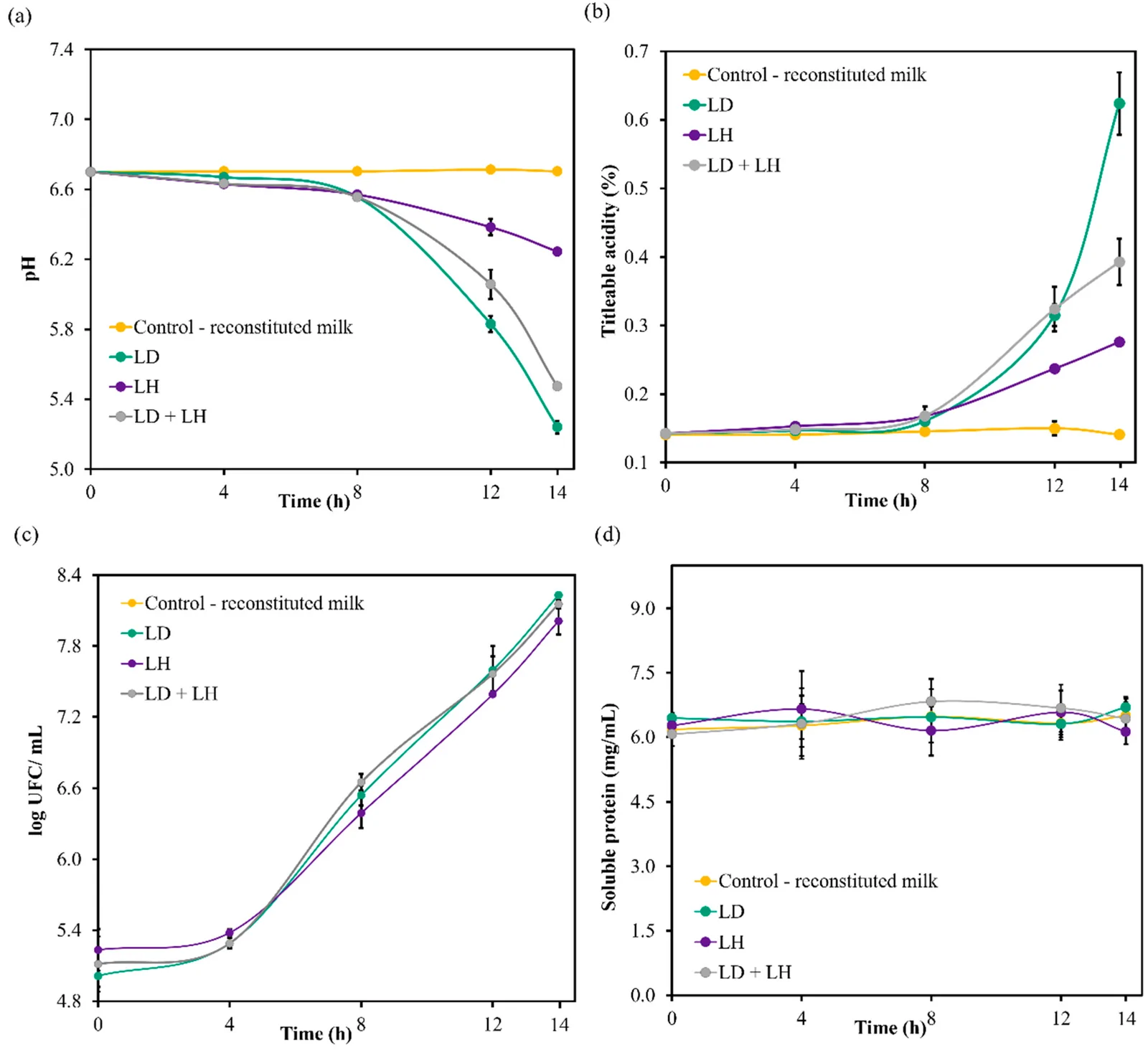
Changes in pH (**a**), titratable acidity (**b**), viable lactic acid bacteria (LAB) counts (**c**), and soluble protein concentration (**d**) in reconstituted milk and fermented milk samples during fermentation (0, 4, 8, 12, and 14 h) at 37 °C. Note: C, reconstituted milk (control); LD, milk fermented with *L. delbrueckii*; LH, milk fermented with *L. helveticus*; LD + LH, milk co-fermented with *L. delbrueckii* and *L. helveticus*. Data are presented as mean ± standard deviation from three independent biological replicates. For each biological replicate, pH, titratable acidity, and soluble protein concentration were determined in triplicate, whereas LAB enumeration was performed in duplicate.

The pH, titratable acidity, viable LAB counts, and soluble protein content of reconstituted milk (control) and fermented milk were monitored at the beginning of fermentation (0 h) and after 4, 8, 12, and 14 h of incubation at 37 °C (Figure 2). The pH of all fermented samples (Figure 2a) decreased progressively throughout fermentation (*p* < 0.05), with the most pronounced decline occurring between 8 and 14 h, reflecting the accumulation of organic acids, predominantly lactic acid, produced during microbial carbohydrate metabolism [53]. At the end of fermentation, pH values ranged from 5.24 ± 0.04 to 6.24 ± 0.02, and the visual appearance of the fermented milk samples is presented in Figure S3 (Supplementary Materials). Among the evaluated strains, LD showed the greatest reduction in pH (21.80%), followed by the co-culture (18.30%) and LH (6.81%) (*p* < 0.05). These differences demonstrate that the strains modified the fermentation environment to different extents under identical experimental conditions. Because acidification directly influences protein organization and enzyme activity during fermentation, these differences may contribute to distinct patterns of protein modification before gastrointestinal digestion [54]. The intermediate pH values observed for the co-culture indicate that combining both strains did not enhance acidification beyond that achieved by LD alone.

Titratable acidity followed the opposite trend (Figure 2b), increasing significantly throughout fermentation (*p* < 0.05), particularly between 8 and 14 h, reaching final values ranging from 0.28 ± 0.01% to 0.62 ± 0.05%. At 14 h, LD exhibited the highest acidification potential (*p* < 0.05), followed by the co-culture and LH, consistent with the corresponding pH profiles. This behavior is attributed to the metabolic activity of LAB, which utilize lactose as their primary carbon source, hydrolyzing it into glucose and galactose and subsequently converting these sugars into lactic acid through the glycolytic pathway [55,56]. The accumulation of lactic acid in the fermentation medium is directly responsible for the increase in titratable acidity [52]. The distinct acidification kinetics observed among the strains indicate differences in their metabolic performance during lactose fermentation under the experimental conditions adopted [57,58]. These differences also resulted in distinct physicochemical environments during fermentation, which may influence protein organization and microbial proteolytic activity before gastrointestinal digestion [59,60]. The accelerated increase in titratable acidity after 8 h coincided with the period of greatest metabolic activity, indicating that most acid production occurred during the later stages of fermentation.

Viable LAB counts (Figure 2c) increased progressively throughout fermentation in all inoculated treatments (*p* < 0.05), rising from 5.02 ± 0.09–5.24 ± 0.18 log CFU/mL at the beginning of incubation to 8.01 ± 0.11–8.23 ± 0.00 log CFU/mL after 14 h. No significant increase in bacterial counts was observed between 0 and 4 h (*p* > 0.05), indicating limited cell multiplication during the initial stage of fermentation. Likewise, no significant differences were detected among LD, LH, and the co-culture throughout fermentation (*p* > 0.05), demonstrating comparable bacterial growth in reconstituted milk. Despite these similar bacterial populations, LD exhibited substantially greater acidification than LH, indicating that fermentation performance depended primarily on strain-specific metabolic characteristics rather than on cell density. As expected, no bacterial growth was detected in the control throughout the experimental period, confirming the effectiveness of the thermal treatment applied before fermentation.

Regarding soluble protein concentration, no statistically significant differences were observed among treatments throughout fermentation (*p* > 0.05), with values ranging from 6.07 ± 0.27 mg/mL to 6.83 ± 0.28 mg/mL (Figure 2d). Although LD and LH possess well-documented proteolytic systems [59], no significant changes in the overall concentration of soluble proteins were detected under the experimental conditions evaluated. This finding does not necessarily indicate the absence of proteolysis but rather suggests that protein hydrolysis occurred to a limited extent and was insufficient to substantially alter the bulk soluble protein fraction. Such behavior is consistent with the mode of action of the CEP system in LAB, which initiates protein hydrolysis through localized cleavage events that primarily generate peptides for bacterial nutrition while preserving much of the protein matrix [61,62,63]. These localized structural modifications are not necessarily reflected by changes in total soluble protein content but may still alter the structural organization of milk proteins, influencing their susceptibility to subsequent gastrointestinal digestion.

### 3.3. Structural Modification and Residual Immunoreactivity of Milk Proteins After Fermentation

**Figure 3:**
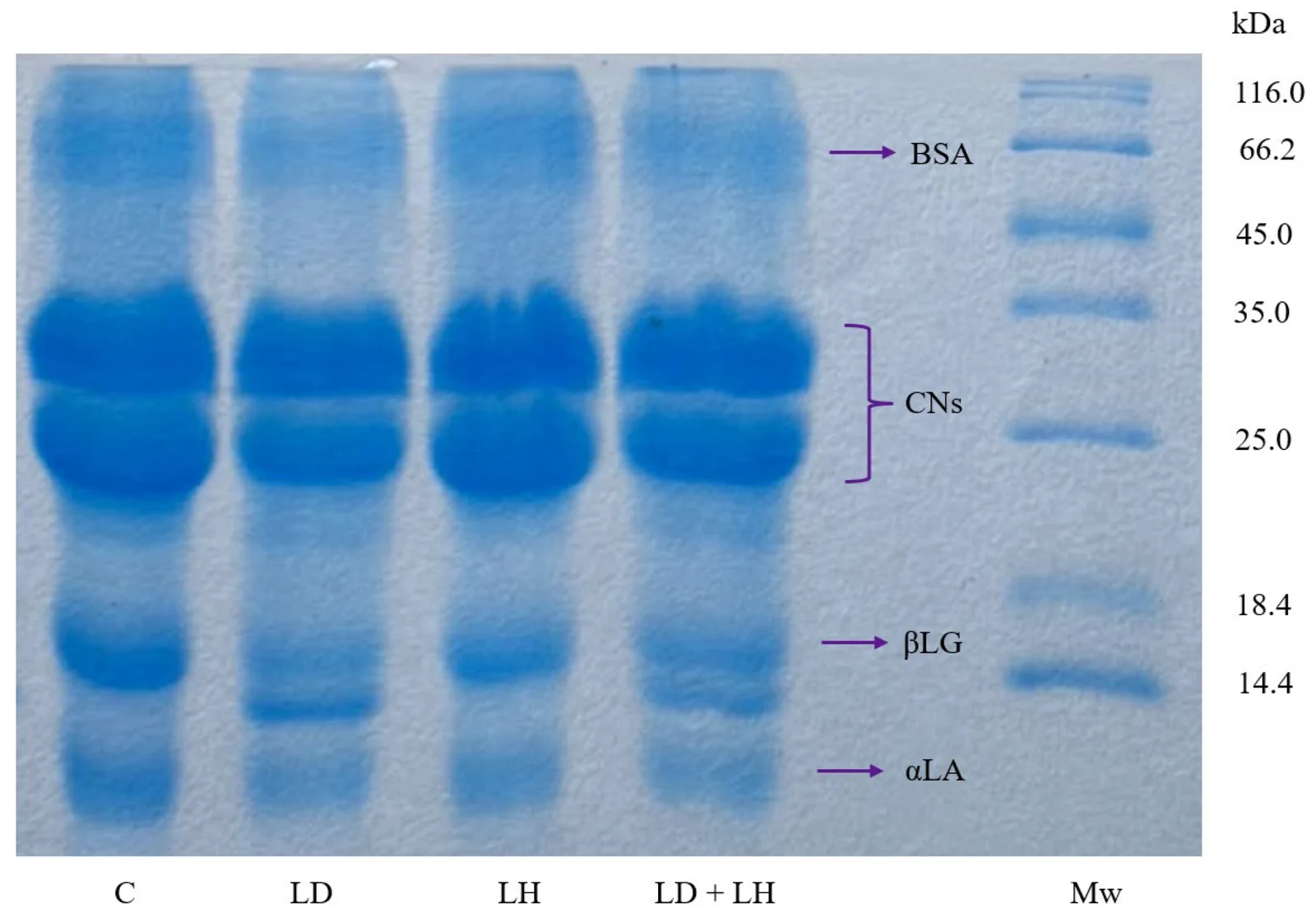
SDS-PAGE profiles of reconstituted milk and fermented milk samples after 14 h of fermentation at 37 °C. Note: Mw, molecular weight marker; C, reconstituted milk (control); LD, milk fermented with *L. delbrueckii*; LH, milk fermented with *L. helveticus*; LD + LH, milk co-fermented with *L. delbrueckii* and *L. helveticus*. SDS-PAGE profiles were qualitatively interpreted based on the visual patterns of the observed protein bands. For each treatment, fermentation was conducted in triplicate, and equal aliquots from independent biological replicates were pooled before SDS-PAGE analysis.

Changes in the protein profile of milk proteins following fermentation were evaluated by SDS-PAGE (Figure 3). Protein bands detected at approximately 25–35 kDa, 14.2 kDa, 18.2 kDa, and 66.2 kDa were consistent with the expected molecular weight ranges of CNs, αLA, βLG, and BSA, respectively, in agreement with previous reports [64,65]. The electrophoretic profiles showed that the major protein fractions remained largely preserved after fermentation regardless of the treatment, indicating limited overall protein degradation under the experimental conditions evaluated. Nevertheless, additional bands were observed in the region between approximately 15 and 20 kDa exclusively in LD and the co-culture, suggesting the formation of intermediate protein fragments during fermentation, as previously reported [12]. Although these changes were not accompanied by extensive degradation of the major protein fractions, they indicate that fermentation was associated with changes in the milk protein profile, particularly in treatments containing LD.

LAB require external nitrogen sources because the concentration of free amino acids naturally present in milk is insufficient to sustain growth. They therefore activate a proteolytic system composed of CEPs, peptide transporters, and intracellular peptidases that sequentially hydrolyze milk proteins into oligopeptides and free amino acids [62,66,67]. The extent of this proteolytic activity depends on the repertoire of enzymes expressed by each strain and is further influenced by environmental factors such as pH, temperature, and nitrogen availability [66,68,69]. The distinct electrophoretic profile observed for LD is consistent with its greater acidification capacity and suggests more extensive protein modification under the fermentation conditions evaluated. Thus, the differences observed among the fermented treatments may reflect not only strain-specific proteolytic activity but also the distinct physicochemical conditions generated during fermentation, particularly the different degrees of acidification [70,71,72]. Importantly, the limited electrophoretic changes observed should not be interpreted as evidence of negligible protein modification [73]. Instead, they indicate that fermentation promoted partial remodeling of the milk protein matrix without extensive degradation of the major protein fractions [62,74]. Such modifications may increase the susceptibility of specific peptide bonds to digestive enzymes, potentially influencing the subsequent gastrointestinal digestion of milk proteins [75,76,77,78].

Residual immunoreactivity of CNs and βLG was evaluated by enzyme-linked immunosorbent assay (ELISA) (Table 2). This analysis complemented the soluble protein and SDS-PAGE results by providing a more sensitive assessment of fermentation-induced protein modifications that are not necessarily detected by changes in electrophoretic profiles alone. βLG was selected as the representative whey protein because it is one of the major allergenic proteins in bovine milk [79,80]. Whereas SDS-PAGE primarily detects changes in molecular weight and the integrity of major protein fractions, ELISA evaluates the persistence of antibody-recognized epitopes, allowing subtle structural modifications associated with proteolysis to be detected even when the overall electrophoretic profile remains largely preserved. Importantly, the changes in ELISA-detectable immunoreactivity observed in this study should not be interpreted as evidence of hypoallergenicity or clinical benefit.

The quantification of ELISA-detectable CNs and βLG further supported the limited but measurable effects of fermentation on milk proteins, complementing the electrophoretic profiles obtained by SDS-PAGE. The different responses observed for CNs and βLG indicate that the susceptibility of milk proteins to microbial proteolysis depends not only on the proteolytic capacity of the bacterial strain but also on the structural characteristics of the protein substrate. Caseins are intrinsically disordered phosphoproteins organized into dynamic micellar structures, which provide greater accessibility of peptide bonds to bacterial cell-envelope proteinases [81,82,83,84]. In contrast, βLG adopts a compact β-barrel globular conformation stabilized by two intramolecular disulfide bonds, limiting enzyme access to susceptible peptide bonds and making this protein considerably more resistant to proteolysis [9,10,11,85]. This structural contrast explains why modifications in CNs were more readily detected than those in βLG, despite exposure to the same fermentation conditions.

The selective reduction in βLG immunoreactivity observed only for LD suggests that strain-dependent differences extend beyond the overall extent of proteolysis and may also reflect differences in substrate specificity of the proteolytic system. Distinct CEP repertoires have been described for different *Lactobacillus* species, resulting in differences in substrate recognition, cleavage preferences, and peptide release patterns during milk fermentation [86,87]. These characteristics may explain why comparable bacterial growth and only modest electrophoretic changes resulted in different immunoreactivity profiles among the fermentation treatments. Differences in the degree of acidification among the strains may also have contributed to the profiles observed, as a reduction in pH can induce changes in the structural characteristics of milk proteins and, consequently, influence their antigenicity [71,88,89]. However, the absence of a non-fermented pH-adjusted control constitutes a limitation of this study.

ELISA detects antibody-recognized epitopes [90] rather than total protein concentration or molecular weight, allowing structural modifications that alter epitope recognition to be identified even when the major protein bands remain largely preserved in SDS-PAGE. The complementary information provided by these two techniques indicates that fermentation promoted localized structural remodeling rather than extensive protein degradation. Such localized modifications are particularly relevant because partial disruption or masking of allergenic epitopes may alter the immunological recognition of milk proteins without requiring complete hydrolysis of the parent protein [91,92,93,94,95]. This observation is consistent with the hypothesis that relatively subtle fermentation-induced structural modifications can influence the subsequent behavior of milk proteins during gastrointestinal digestion.

The combined SDS-PAGE and ELISA results indicate that fermentation-induced protein modifications were more pronounced in LD-treated samples. The absence of detectable changes in milk fermented with LH may reflect differences in the specificity, expression, and catalytic efficiency of cell-envelope proteinases (CEPs), as well as differences in acidification and strain-dependent responses to the fermentation conditions [63,70,96]. Although the reductions in immunoreactivity were modest, they demonstrate that fermentation promoted selective structural modifications without extensive degradation of the major milk protein fractions. These modifications are particularly relevant because changes in protein structure, even when limited, can alter enzyme accessibility and subsequent proteolytic susceptibility, providing a mechanistic link between fermentation and the gastrointestinal digestion behavior evaluated in the following experiments. This interpretation is consistent with the central hypothesis of the present study, namely that strain-dependent fermentation modifies milk proteins before digestion and thereby influences their subsequent gastrointestinal behavior.

### 3.4. Protein Digestion Profiles Under Age-Specific in Vitro Gastrointestinal Conditions

**Figure 4:**
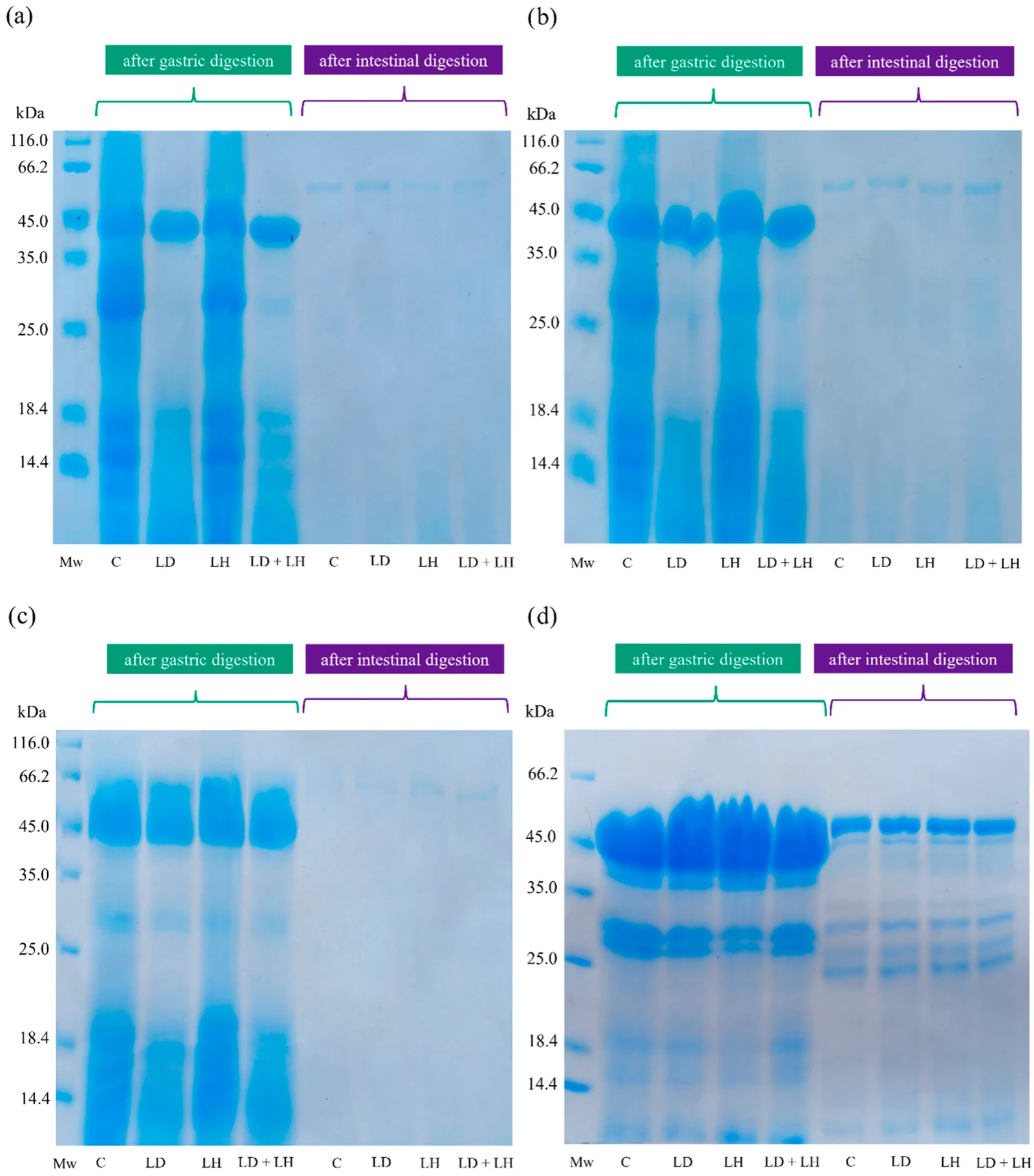
SDS-PAGE profiles of reconstituted milk and fermented milk samples after in vitro gastrointestinal digestion under age-specific digestion models representing (**a**) 1-month-old infants, (**b**) 3-month-old infants, (**c**) 6-month-old infants, and (**d**) adults. Electrophoretic profiles were obtained after the gastric and intestinal digestion phases. Note: Mw, molecular weight marker; C, reconstituted milk (control); LD, milk fermented with *L. delbrueckii*; LH, milk fermented with *L. helveticus*; LD + LH, milk co-fermented with *L. delbrueckii* and *L. helveticus*. SDS-PAGE profiles were qualitatively interpreted based on the visual patterns of the observed protein bands. Digestion experiments were performed using two independent biological replicates, and equal aliquots from the biological replicates were pooled before SDS-PAGE analysis.

Reconstituted milk and fermented milk samples were subjected to in vitro gastrointestinal digestion models representative of 1-, 3-, and 6-month-old infants and adults (Figure 4). SDS-PAGE was used to evaluate how fermentation influenced the susceptibility of milk proteins to gastrointestinal proteolysis under physiological conditions representative of different stages of digestive maturity. Comparing the digestion profiles of fermented and non-fermented milk qualitatively made it possible to determine whether the structural modifications induced during fermentation persisted throughout digestion and whether these effects were influenced by age-specific gastrointestinal conditions.

Figure 4a,b show the SDS-PAGE profiles obtained after simulated in vitro gastrointestinal digestion using models representative of 1- and 3-month-old infants, respectively. During the gastric phase, a visually greater reduction in bands within the molecular weight regions expected for CN and whey proteins was observed in milk fermented with LD and, to a lesser extent, in the co-culture, suggesting greater susceptibility of milk proteins to gastric proteolysis after fermentation. The partial structural remodeling promoted during fermentation appears to have facilitated pepsin access to susceptible peptide bonds, particularly those adjacent to aromatic and hydrophobic amino acid residues, including tryptophan, tyrosine, phenylalanine, leucine, and isoleucine [8,97,98,99]. Rather than promoting extensive protein degradation before digestion, fermentation-induced modifications in the protein matrix appeared to increase the susceptibility of milk proteins to hydrolysis during the gastric phase.

The persistence of intact proteins and high-molecular-weight fragments, observed as diffuse staining in the lower region of the gel, is consistent with the digestive physiology of infants at these ages, which is characterized by higher gastric pH and lower pepsin activity than in adults [12,44,100,101]. From birth to approximately 3 months of age, pepsin secretion increases progressively but remains substantially lower than adult levels, resulting in incomplete gastric proteolysis and a greater proportion of partially hydrolyzed proteins reaching the intestinal phase [44,102]. This physiological context makes the effects of microbial pre-treatment more evident, as even subtle structural modifications introduced during fermentation can increase the susceptibility of milk proteins to gastric digestion when endogenous proteolytic capacity is limited. A protein band between 35 and 45 kDa, consistent with the expected molecular weight range of pepsin, was detected after the gastric phase, as previously reported [103,104,105].

The SDS-PAGE profiles obtained using the 6-month-old infant digestion model are shown in Figure 4c. During the gastric phase, a visually more pronounced reduction in bands within the molecular weight regions expected for CN was observed across all treatments, including the control, compared with the 1- and 3-month-old infant models. This behavior reflects the progressive maturation of the gastric digestive system, characterized by increased pepsin activity and lower gastric pH at this stage of development [106,107]. The greater efficiency of endogenous gastric proteolysis reduced the differences among the fermentation treatments, indicating that the contribution of microbial pre-treatment became less pronounced as digestive capacity increased. Although fermentation-induced structural modifications were still present, their relative impact on protein hydrolysis was diminished under the more favorable gastric conditions of the 6-month-old model. The influence of fermentation became less evident as digestive maturity increased, indicating that endogenous gastric proteolysis progressively became the dominant factor governing protein hydrolysis [106].

During the gastric phase of the adult digestion model (Figure 4d), bands between 35 and 45 kDa, consistent with the expected molecular weight range of pepsin, were more visually intense because of the higher enzyme concentration used to reproduce adult physiological conditions, characterized by greater pepsin secretion and lower gastric pH than those of infants [12,43,107]. Despite the greater gastric proteolytic capacity, bands compatible with CN remained detectable after the gastric phase, indicating that gastric digestion alone was insufficient to completely hydrolyze these proteins. This observation is consistent with the physiological sequence of protein digestion, in which gastric proteolysis initiates protein breakdown and generates intermediate protein fragments that are further hydrolyzed during the intestinal phase. The less pronounced differences among the fermentation treatments compared with the infant models further indicate that, under adult physiological conditions, gastric proteolysis became the predominant determinant of protein hydrolysis, reducing the relative contribution of fermentation-induced structural modifications.

The whey protein bands exhibited lower visual intensity after the gastric phase in the adult digestion model than in the infant models (Figure 4a–d), reflecting the greater proteolytic capacity associated with adult gastric conditions. Nevertheless, these proteins remained detectable after gastric digestion, confirming their intrinsic resistance to pepsin hydrolysis, as previously reported [78,102,108]. This behavior was particularly evident for bands in the molecular weight range expected for βLG, whose compact globular structure, rich in β-sheets and stabilized by intramolecular disulfide bonds, restricts enzyme access to susceptible peptide bonds [8,9,11]. The persistence of this band across all digestion models suggests that the corresponding protein remained detectable after gastric digestion, even after fermentation [109,110]. Instead, fermentation appeared to increase the susceptibility of the more readily hydrolyzed protein fractions to gastrointestinal proteolysis, whereas the band consistent with βLG became less visually intense during the intestinal phase, suggesting further hydrolysis by the combined action of gastric and pancreatic proteases [78,107]. This finding is particularly relevant for bovine milk-based infant formulas, in which the persistence of partially digested βLG may prolong the exposure of immunoreactive epitopes within the gastrointestinal tract [111,112,113].

During the intestinal phase of both the infant and adult digestion models (Figure 4a–d), a marked reduction in the visual intensity of protein bands was observed across all treatments, consistent with extensive protein hydrolysis. The greater efficiency of intestinal digestion substantially reduced the differences previously observed after the gastric phase, suggesting that pancreatic proteolysis became the predominant determinant of the observed protein profiles regardless of the fermentation treatment. The effects of fermentation were therefore most evident during gastric digestion, whereas intestinal proteolysis progressively minimized these differences. Additional bands, more evident in the adult model because of the higher concentration of pancreatin used to reproduce adult physiological conditions, were observed and may be attributed to proteins present in the pancreatin preparation, as previously reported [8,114,115].

The digestion profiles indicate that the effects of fermentation depended on digestive maturity. Under infant physiological conditions, the structural modifications introduced during fermentation were more evident because endogenous proteolysis was still limited [44,111]. As digestive capacity increased, these differences became less pronounced, and the digestion profiles were progressively determined by the physiological action of the digestive enzymes.

### 3.5. In Silico Prediction of Milk Protein Hydrolysis

**Figure 5:**
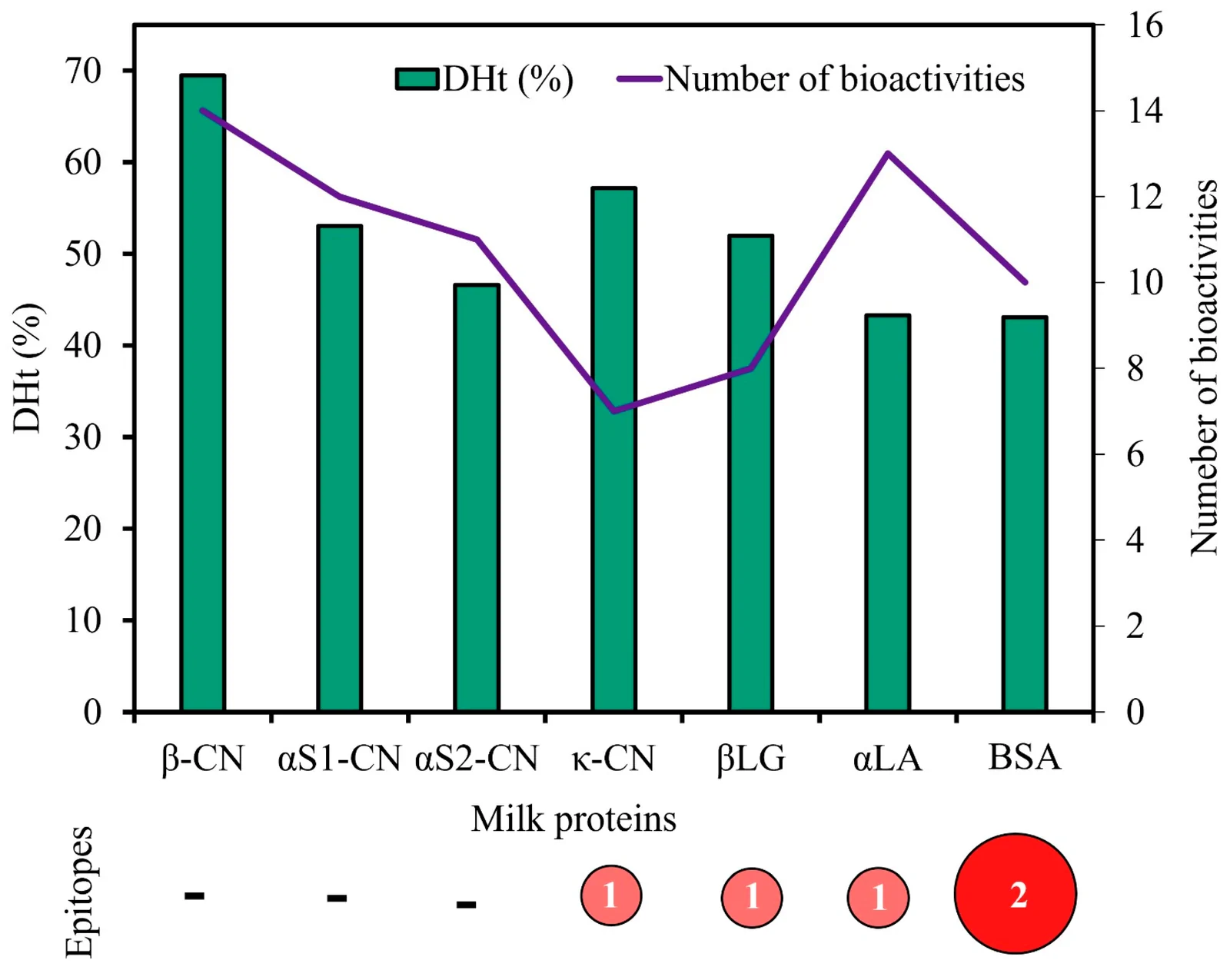
In silico hydrolysis of major bovine milk proteins by lactocepin, showing the theoretical degree of hydrolysis (DHt), predicted bioactive peptide activities, and predicted residual linear allergenic epitope sequences. Note: DHt, theoretical degree of hydrolysis. The purple lines represent the number of predicted bioactive peptide activities identified in BIOPEP-UWM after simulated lactocepin hydrolysis. The red circles indicate the number of potential linear allergenic epitope sequences predicted to remain after simulated lactocepin hydrolysis. These values represent theoretical predictions based on the presence of linear epitope sequences and should not be interpreted as a direct measure of residual allergenicity or as evidence of retained allergenic activity. β-CN, β-casein; αS1-CN, αS1-casein; αS2-CN, αS2-casein; κ-CN, κ-casein; βLG, β-lactoglobulin; αLA, α-lactalbumin; BSA, bovine serum albumin.

To complement the experimental analyses, potential milk protein hydrolysis patterns were explored in silico using lactocepin as a representative model of the major cell-envelope proteinase activity associated with LH and LD (Figure 5). In LH, five CEPs have been described, among which PrtH2, encoded by the *prtH2* gene, is considered ubiquitous among the strains characterized to date [116,117,118]. In LD, the best-characterized CEP is PrtB, encoded by the *prtB* gene and considered species-specific [119]. Both enzymes belong to the lactocepin family (EC 3.4.21.96) and are classified as serine endopeptidases.

The theoretical degree of hydrolysis (DHt) of the major bovine milk proteins was predicted using the BIOPEP-UWM database according to the reported cleavage specificity of lactocepins, which preferentially hydrolyze peptide bonds at the C-terminal side of Q, V, P, and L residues and at the N-terminal side of P, Q, S, and L residues. Predicted DHt values ranged from 43.07% to 69.50%. β-CN exhibited the highest predicted DHt (69.50%), followed by κ-CN (57.14%), αS1-CN (53.05%), βLG (51.91%), and αS2-CN (46.61%), whereas BSA (43.07%) and αLA (43.26%) showed the lowest predicted susceptibility to hydrolysis. The higher DHt predicted for β-CN is consistent with the greater abundance of lactocepin recognition sites within its primary sequence, resulting in a larger number of potential cleavage sites. This prediction is qualitatively consistent with the experimental observations, in which the casein fraction showed greater evidence of fermentation-associated modification than the globular whey proteins, particularly βLG.

Regardless of the predicted DHt, the simulated hydrolysis predicted the release of peptides with potential biological activities from all seven milk proteins evaluated. The predicted peptide sequences and their respective predicted biological activities are presented in Table S2 (Supplementary Materials). β-CN generated the greatest diversity of predicted bioactivities (n = 14), followed by αLA (n = 13) (Figure 5). The in silico hydrolysis predicted no remaining potential linear allergenic epitope sequences for β-CN, αS1-CN, and αS2-CN after simulated lactocepin hydrolysis. In contrast, potential allergenic epitopes were predicted for κ-CN, βLG, αLA, and BSA. The remaining epitope sequences corresponded to IRCEKDERFF (κ-CN, residues 30–39), KKIIAEKTKI (βLG, residues 85–94), INNKIWCKDD (αLA, residues 74–83), and two BSA-derived epitopes, CDEFKADEKKFWGKY (residues 147–161) and EYAV (residues 363–366). These results indicate that the predicted persistence of linear allergenic epitope sequences depends not only on the overall extent of protein hydrolysis but also on whether enzymatic cleavage occurs within or adjacent to these sequences [91,93,94]. As a result, proteins with similar theoretical degrees of hydrolysis may differ substantially in their residual immunoreactivity.

Bovine milk proteins may contain both linear and conformational epitopes [120]. Linear epitopes consist of continuous amino acid sequences, whereas conformational epitopes depend on the three-dimensional organization of the protein, which brings non-consecutive residues in the primary sequence into proximity [121]. The integrity of linear epitopes may influence their recognition by the immune system and trigger an immune response [122]. However, the predicted reduction in the number of linear epitope sequences in the present study should not be interpreted as complete elimination of allergenic potential. The in silico analysis performed in this study considers linear epitope sequences and the reported cleavage specificity of lactocepin but does not account for the three-dimensional structure of the proteins or conformational changes that may occur during fermentation. These changes may alter the exposure of conformational epitopes, making them more or less accessible to immune recognition [123].

The in silico results represent theoretical predictions based exclusively on the cleavage specificity of lactocepin. Under in vitro conditions, the actual extent of hydrolysis may be lower than predicted [124], particularly for whey proteins such as βLG, αLA, and BSA, whose compact globular structures and intramolecular disulfide bonds restrict enzyme access to susceptible peptide bonds [9,125]. This is consistent with the experimental results obtained in the present study, in which βLG exhibited only a modest reduction in immunoreactivity after fermentation despite its predicted susceptibility to hydrolysis. The differences between the predicted and experimental results indicate that cleavage specificity alone does not determine the extent of protein hydrolysis under biological conditions [126,127]. Protein conformation also plays an important role by limiting enzyme accessibility [128,129]. In silico analysis is useful for predicting potential cleavage events but does not fully represent protein behavior under experimental conditions. Native protein structure remains an important determinant of enzyme accessibility.

## 4. Conclusions

LD and LH exhibited distinct acidification patterns and fermentation-associated protein modifications during milk fermentation. LD showed the greatest acidification capacity and was the only treatment that significantly reduced residual βLG immunoreactivity, while both LD and the co-culture decreased the ELISA-detectable CN fraction, although these reductions were modest. These fermentation-induced modifications were associated with increased susceptibility of milk proteins to gastric digestion, particularly under physiological conditions representative of early infancy, whereas their influence progressively declined as digestive maturity increased. Throughout the digestion models, bands compatible with βLG remained among the most resistant milk protein bands, highlighting the combined influence of protein structure and digestive physiology on gastrointestinal proteolysis. The in silico analysis complemented the experimental results by predicting preferential hydrolysis of caseins, the release of peptides with potential biological activities, and the differential persistence of linear allergenic epitope sequences among milk proteins. Together, the experimental and computational results indicate that the effects of fermentation depend not only on strain-specific protein modifications but also on the physiological conditions under which digestion occurs. Although fermentation alone produced only modest reductions in residual immunoreactivity, it modified the subsequent digestive behavior of milk proteins, particularly under infant physiological conditions. These findings contribute to the understanding of how fermentation-induced protein modifications and digestive maturity interact and provide a basis for further investigation of fermented dairy systems under early-life physiological conditions. Further studies are needed to determine whether these molecular modifications translate into reduced allergenicity and measurable biological and clinical benefits. These conclusions should therefore be interpreted within the context of the processed reconstituted milk system evaluated in this study.

## Figures and Tables

**Table 1:** Taxonomic identification of the bacterial microorganisms based on 16S rRNA gene sequencing and comparison with reference sequences deposited in the NCBI GenBank database.

Taxonomic Classification	*Lactobacillus delbrueckii*	*Lactobacillus helveticus*
NCBI taxonomic classification		
Domain	Bacteria	Bacteria
Kingdom	Bacillati	Bacillati
Phylum	Bacillota	Bacillota
Class	Bacilli	Bacilli
Order	Lactobacillales	Lactobacillales
Family	Lactobacillaceae	Lactobacillaceae
Genus	*Lactobacillus*	*Lactobacillus*
Species	*delbrueckii*	*helveticus*
Sequence similarity (%)	99.20	99.29
Query coverage	100% (627 bp)	100% (1417 bp)

Note: The identification was performed using one sample of each bacterial strain.

**Table 2:** Residual ELISA-detectable caseins (CNs) and β-lactoglobulin (βLG) in reconstituted milk and fermented milk samples, and their percentage reduction relative to the control.

Samples	Residual CNs Detected by ELISA (mg/kg)	Reduction in Detectable CNs (%)	Residual βLG Detected by ELISA (mg/kg)	Reduction in Detectable βLG (%)
Control—reconstituted milk	18,975 ± 106 ^a^	NA	8.41 ± 0.02 ^a^	NA
LD	17,567 ± 45 ^b^	7.42 ± 0.28 ^a^	8.19 ± 0.01 ^b^	2.17 ± 0.20 ^a^
LH	19,072 ± 180 ^a^	NR	8.41 ± 0.01 ^a^	0.08 ± 0.35 ^b^
LD + LH	17,295 ± 77 ^b^	8.85 ± 0.92 ^a^	8.41 ± 0.01 ^a^	0.03 ± 0.05 ^b^

Note: C, reconstituted milk (control); LD, milk fermented with *L. delbrueckii*; LH, milk fermented with *L. helveticus*; LD + LH, milk co-fermented with *L. delbrueckii* and *L. helveticus*. Results are expressed as mean ± standard deviation (SD) from two independent biological replicates. Different lowercase letters within the same column indicate significant differences among treatments according to Tukey’s test (*p* ≤ 0.05), whereas the same lowercase letters indicate no significant differences (*p* > 0.05). NA, not applicable (the control sample was used as the reference for percentage reduction); NR, no reduction relative to the control.

## Data Availability

The raw data supporting the conclusions of this article will be made available by the authors upon request.

## Supplementary Materials

The following supporting information can be downloaded at https://www.mdpi.com/article/10.3390/foods15203712/s1, Table S1: Milk protein sequences and their respective allergenic epitopes used in in silico enzymatic hydrolysis simulations, obtained from the BIOPEP-UWM platform; Figure S1: Growth profile of *L. delbrueckii* and *L. helveticus* strains, determined by optical density (OD_600_), in MRS broth inoculated with 1% (*v*/*v*) of the bacterial culture. Data are presented as mean ± standard deviation of triplicate measurements.; Figure S2: Phenetic tree showing the phylogenetic distance between the acquired bacterial strains based on 16S rRNA. Constructed using MEGA 11 software via the Neighbor-Joining method with the Jukes–Cantor parameter and 1000 bootstrap replicates. The identification was performed using one sample of each bacterial strain.; Figure S3: Visual appearance of reconstituted milk and fermented milk samples. C: reconstituted milk (control); LD: fermented milk with *L. delbrueckii*; LH: fermented milk with *L. helveticus*; LD + LH: fermented milk with *L. delbrueckii* and *L. helveticus*. Images were obtained from pooled samples prepared from triplicate biological replicates.; Table S2: Simulated enzymatic hydrolysis of bovine milk proteins by the enzyme lactocepin (EC 3.4.21.96) using the BIOPEP-UWM platform.
