# Supplementary material for: Strain-Dependent Lactic Fermentation Primes Milk Proteins for Gastrointestinal Digestion Under Age-Specific Physiological Conditions
Source: Foods. 2026 Oct 19;15(20):3712. doi: 10.3390/foods15203712 (PMC13704181; doi:10.3390/foods15203712)
Supplement: Supplementary file 1 [file foods-15-03712-s001.zip › foods-4569809-supplementary.pdf]

SUPPLEMENTARY MATERIAL

Table S1: Milk protein sequences and their respective allergenic epitopes used in *in silico* enzymatic hydrolysis simulations, obtained from the BIOPEP-UWM platform.

Protein	Protein sequence	Lenght	Mass (KDa)	Structure of epitopes
β-CN	MKVLILACLVALALARELEELNVPGEIVESLSSSEESITRINKKIEKFQSEEQQQTEDELQDKIHPFAQTQSLVYPFPGPIPNSLPQNIPPLTQTPVVVPPFLQPEVMGVSKVKEAMAPKHKEMPFPKYPVEPFTESQSLTLTDVENLHLPLPLLQSWMHQPHQPLPPTVMFPPQSVLSLSQSKVLPVPQKAVPYPQRDMPIQAFLLYQEPVLGPVRGPFPIIV	224	25.11	RELEELNVPG,RELEELNVPGEIVE,RELEELNVPGEIVESL,RELEELNVPGEIVESLSSSE,LEELNVPGEI,EELNVPGEIVESLSSSEESI,ELNVPGEIVE,NVPGEIVESL,NVPGEIVESLSSSEESITRI,PGEIVESLSS,GEIVESLSSSEESITRINKK,EIVESLSSSE,VESLSSSEES,VESLSSSEESITRINKKIEK,SLSSSEESIT,LSSSEESITRINKKIEKFQS,SSSEESITRI,SEESITRINK,SEESITRINKKIEKFQSEEQ,ESITRINKKI,SITRINKKIEKFQSEEQQQT,ITRINKKIEK,RINKKIEKFQ,RINKKIEKFQSEEQQQTEDE,RINKKIEKFQSEEQQQTEDELQDKIH,NKKIEKFQSE,KKIEKFQSEEQQQTEDELQD,KIEKFQSEEQ,EKFQSEEQQQ,EKFQSEEQQQTEDELQDKIH,FQSEEQQQTE,QSEEQQQTEDELQDKIHPFA,SEEQQQTEDE,EQQQTEDELQ,EQQQTEDELQDKIHPFAQTQ,QQTEDELQDK,QTEDELQDKIHPFAQTQSLV,TEDELQDKIH,DELQDKIHPF,DELQDKIHPFAQ,DELQDKIHPFAQTQSLVYPF,LQDKIHPFAQ,QDKIHPFAQTQSLVYPFPGP,DKIHPFAQTQ,IHPFAQTQSL,IHPFAQTQSLVYPFPGPIPN,PFAQTQSLVY,FAQTQSLVYPFPGPIPNSLP,FAQTQSLVYPFPGPIPNSLPQNI,AQTQSLVYPF,TQSLVYPFPG,TQSLVYPFPGPIPNSL,TQSLVYPFPGPIPNSLPQNI,SLVYPFPGPI,SLVYPFPGPIPNSL,LVYPFPGPIPNSLPQNIPPL,VYPFPGPIPN,PFPGPIPNSL,PFPGPIPNSLPQNIPPLTQT,PGPIPNSLPQ,GPIPNSLPQNIPPLTQTPVV,PIPNSLPQNI,PNSLPQNIPPLTQTPVVVPP,PNSLPQNIPP,SLPQNIPPLT,LPQNIPPLTQTPVVVPPFLQ,PQNIPPLTQT,NIPPLTQTPVVVPPFLQPEV,NIPPLTQTPV,PPLTQTPVVV,PLTQTPVVVPPFLQPEVMGV,LTQTPVVVPP,QTPVVVPPFL,QTPVVVPPFLQPEVMGVSKV,PVVVPPFLQP,VVVPPFLQPEVMGVSKVKEA,VVPPFLQPEV,PPFLQPEVMG,PPFLQPEVMGVSKVKEAMAP,FLQPEVMGVS,LQPEVMGVSKVKEAMAPKHK,QPEVMGVSKV,EVMGVSKVKE,EVMGVSKVKEAMAPKHKEMP,MGVSKVKEAM,GVSKVKEAMAPKHKEMPFPK,VSKVKEAMAP,KVKEAMAPKH,KVKEAMAPKHKEMPFPKYPV,KEAMAPKHKE,EAMAPKHKEMPFPKYPVEPF,AMAPKHKEMP,APKHKEMPFP,APKHKEMPFPKYPVEPFTES,KHKEMPFPKY,HKEMPFPKYPVEPFTESQSL,KEMPFPKYPV,KEMPFPKYPVEPFT,MPFPKYPVEP,MPFPKYPVEPFTESQSLTLT,FPKYPVEPFT,PKYPVEPFTESQSLTLTDVE,KYPVEPFTES,PVEPFTESQS,PVEPFTESQSLTLTDVENLH,EPFTESQSLT,PFTESQSLTLTDVENLHLPL,FTESQSLTLT,ESQSLTLTDV,ESQSLTLTDVENLHLPLPLL,QSLTLTDVEN,SLTLTDVENLHLPLPLLQSW,LTLTDVENLH,LTDVENLHLP,LTDVENLHLPLPLLQSWMHQ,DVENLHLPLP,VENLHLPLPLLQSWMHQPHQ,ENLHLPLPLL,LHLPLPLLQS,LHLPLPLLQSWMHQPHQPLP,LPLPLLQSWM,PLPLLQSWMHQPHQPLPPTV,LPLLQSWMHQ,LLQSWMHQPH,LLQSWMHQPHQPLPPTVMFP,QSWMHQPHQP,SWMHQPHQPLPPTVMFPPQS,WMHQPHQPLP,HQPHQPLPPT,HQPHQPLPPTVMFPPQSVLS,PHQPLPPTVM,HQPLPPTVMFPPQSVLSLSQ,QPLPPTVMFP,QPLPPTVMFPPQ,QPLPPTVMFPPQSVLS,LPPTVMFPPQ,LPPTVMFPPQSVLSLSQSKV,PTVMFPPQSV,TVMFPPQSVLSLSQSKVLPV,VMFPPQSVLS,FPPQSVLSLS,FPPQSVLSLSQSKVLPVPQK,PQSVLSLSQS,QSVLSLSQSKVLPVPQKAVP,SVLSLSQSKV,LSLSQSKVLP,LSLSQSKVLPVPQKAVPYPQ,LSQSKVLPVP,SQSKVLPVPQKAVPYPQRDM,QSKVLPVPQK,QSKVLPVPQKAV,QSKVLPVPQKAVPYPQRD,KVLPVPQKAV,KVLPVPQKAVPYPQRDMPIQ,LPVPQKAVPY,PVPQKAVPYPQRDMPIQAFL,VPQKAVPYPQ,VPQKAVPYPQRD,QKAVPYPQRD,QKAVPYPQRDMPIQAFLLYQ,AVPYPQRDMP,VPYPQRDMPIQAFLLYQEPV,PYPQRDMPIQ,PQRDMPIQAF,PQRDMPIQAFLLYQEPVLGP,RDMPIQAFLL,DMPIQAFLLYQEPVLGPVRG,MPIQAFLLYQ,MPIQAFLLYQEP,MPIQAFLLYQEPVLGPVRGPFPII,IQAFLLYQEP,IQAFLLYQEPVLGPVRGPFP,AFLLYQEPVL,FLLYQEPVLGPVRGPFPIIV,LLYQEPVLGP,YQEPVLGPVR,YQEPVLGPVRGPFP,EPVLGPVRGP,VLGPVRGPFP,GPVRGPFPII,CLVALALARELEELNVPGEIVESLSSSEESI,LTQTPVVVPPFLQPEVMGVSKV,VVVPPFLQPEVMGVSKVKEAMAPKHKEMPFP,GVSKVKEAMAPKHKEMPFPKYP,FPKYPVEPFTESQSLTLTDVENL
αS1-CN	MKLLILTCLVAVALARPKHPIKHQGLPQEVLNENLLRFFVAPFPEVFGKEKVNELSKDIGSESTEDQAMEDIKQMEAESISSSEEIVPNSVEQKHIQKEDVPSERYLGYLEQLLRLKKYKVPQLEIVPNSAEERLHSMKEGIHAQQKEPMIGVNQELAYFYPELFRQFYQLDAYPSGAWYYVPLGTQYTDAPSFSDIPNPIGSENSEKTTMPLW	214	24.53	RPKHPIKHQG,RPKHPIKHQGLPQEVLNE,RPKHPIKHQGLPQEVLNENL,RPKHPIKHQGLPQEVLNENLLRFFVAPFPEV,KHPIKHQGLP,HPIKHQGLPQEVLNENLLRF,PIKHQGLPQE,KHQGLPQEVLNENLLRFF,KHQGLPQEVLNENLLRFFVA,QGLPQEVLNE,GLPQEVLNENLLRFFVAPFP,LPQEVLNENL,QEVLNENLLR,QEVLNENLLRFFVAPFPE,QEVLNENLLRFFVAPFPEVF,VLNENLLRFF,VLNENLLRFFVAPFPEVFGKEK,VLNENLLRFFVAPFPEVFGKEKVNELSKDIGS,LNENLLRFFVAPFPEVFGKE,NENLLRFFVA,NENLLRFFVAPFPE,NENLLRFFVAPFPEVFGKEK,NLLRFFVAPF,NLLRFFVAPFPE,NLLRFFVAPFPEVFGKEK,NLLRFFVAPFPEVFGKEKVN,LLRFFVAPFPEV,LRFFVAPFPE,RFFVAPFPEVFGKEKVNELS,FFVAPFPEVF,FFVAPFPEVFGKEK,VAPFPEVFGK,VAPFPEVFGKEK,VAPFPEVFGKEKVNELSK,VAPFPEVFGKEKVNELSKDI,PFPEVFGKEK,FPEVFGKEKVNELSKDIGSE,FPEVFGKEKVNELSKDIGSESTE,PEVFGKEKVN,VFGKEKVNEL,VFGKEKVNELSKDIGSESTE,VFGKEKVNELSKDIGSES,FGKEKVNELSKDIGSESTEDQAMEDIKQMEAES,GKEKVNELSK,KEKVNELSKDIG,KEKVNELSKDIGSESTEDQA,EKVNELSKDI,VNELSKDIGS,VNELSKDIGSESTEDQAM,VNELSKDIGSESTEDQAMED,ELSKDIGSES,LSKDIGSESTEDQAMEDIKQ,SKDIGSESTE,DIGSESTEDQ,DIGSESTEDQAMEDIKQM,DIGSESTEDQAMEDIKQMEA,GSESTEDQAM,SESTEDQAMEDIKQMEAE,SESTEDQAMEDIKQMEAESI,ESTEDQAMED,TEDQAMEDIKQMEAESIS,TEDQAMEDIKQMEAESIS,TEDQAMEDIKQMEAESISSS,TEDQAMEDIK,EDQAMEDIKQMEAESISSSEEIVP,EDQAMEDIKQMEAESISSSEEIVPNSVEQK,DQAMEDIKQM,QAMEDIKQMEAESISSSEEI,AMEDIKQMEA,EDIKQM,EDIKQMEAES,EDIKQMEAESIS,EDIKQMEAESISSSEEIV,EDIKQMEAESISSSEEIVPN,IKQMEAESIS,KQMEAESISSSEEIVP,KQMEAESISSSEEIVPNSVE,QMEAESISSS,EAESISSSEE,EAESISSSEEIVPNSVE,EAESISSSEEIVPNSVEQ,EAESISSSEEIVPNSVEQKH,ESISSSEEIV,SISSSEEIVPNSVEQKHIQK,ISSSEEIVPN,ISSSEEIVPNSVEQKHIQKEDVPSERYLGYLEQLLR,SSEEIVPNSV,SSEEIVPNSVEQKHIQKE,SSEEIVPNSVEQKHIQKEDV,EEIVPNSVEQ,EIVPNSVEQKHIQKEDVPSE,IVPNSVEQKH,PNSVEQKHIQ,PNSVEQKHIQKEDVPSER,PNSVEQKHIQKEDVPSERYL,SVEQKHIQKE,VEQKHIQKEDVPSERYLGYL,EQKHIQKEDV,KHIQKEDVPS,KHIQKEDVPSER,KHIQKEDVPSERYLGYLE,KHIQKEDVPSERYLGYLEQL,IQKEDVPSER,QKEDVPSERYLGYLEQLLRL,KEDVPSERYL,KEDVPSERYLGYLEQLLRLK,DVPSERYLGY,DVPSERYLGYLEQLLRLK,DVPSERYLGYLEQLLRLKKY,VPSERYLGYLEQLLRLKK,VPSERYLGYLEQLLRLKKYKVPQLEIVPNS,PSERYLGYLE,SERYLGYLEQLLRLKKYKVP,ERYLGYLEQL,ERYLGYLEQLLRLK,YLGYLEQLLRLKKYKVPQ,YLGYLEQLLRLKKYKVPQLE,GYLEQL,GYLEQLLRLK,GYLEQLLRLKKYKVPQ,YLEQLLRLKKYKVPQLEIVP,LEQLLRLKKY,QLLRLKKYKV,QLLRLKKYKVPQLEIVPN,QLLRLKKYKVPQLEIVPNSA,LRLKKYKVPQ,RLKKYKVPQLEIVPNSAEER,LKKYKVPQLE,LKKYKVPQLEIVPNSAEERLHSMKEGIHAQQKE,KYKVPQLEIV,KYKVPQLEIVPNSAEERL,KYKVPQLEIVPNSAEERLHS,KVPQLEIVPN,VPQLEIVPNSAEERLHSMKE,PQLEIVPNSA,LEIVPNSAE,LEIVPNSAEE,LEIVPNSAEERL,LEIVPNSAEERLHSMKEG,LEIVPNSAEERLHSMKEGIH,IVPNSAEERL,VPNSAEERLHSMKEGIHAQQ,PNSAEERLHS,SAEERLHSMK,SAEERLHSMKEGIHAQQK,SAEERLHSMKEGIHAQQKEP,EERLHSMKEG,ERLHSMKEGIH

				AQQKEPMIG,RLHSMKEGIH,RLHSMKEGIHAQQKEPMIGVNQELAYFYPE,HSMKEGIHAQ,HSMKEGIHAQQKEPMIGV,HSMKEGIHAQQKEPMIGVNQ,MKEGIHAQQK,KEGIHAQQKEPM,KEGIHAQQKEPMIGVNQELA,EGIHAQQKEP,IHAQQKEPMI,IHAQQKEPMIGVNQELAY,IHAQQKEPMIGVNQELAYFY,AQQKEPMIGV,QQKEPMIGVNQELAYFYPEL,QKEPMIGVNQ,EPMIGVNQEL,EPMIGVNQELAY,EPMIGVNQELAYFYPELF,EPMIGVNQELAYFYPELFRQ,PMIGVNQELAYFYPELFRQFYQLDAYPSGAWYYV,MIGVNQELAY,IGVNQELAYFYPELFRQFYQ,GVNQELAYFY,NQELAYFYPE,NQELAYFYPELF,NQELAYFYPELFRQ,NQELAYFYPELFRQF,NQELAYFYPELFRQFY,NQELAYFYPELFRQFYQL,NQELAYFYPELFRQFYQLDA,ELAYFYPELF,LAYFYPELFRQFYQLDAYPS,AYFYPELFRQ,AYFYPELFRQFYQLDAYP,YLGYLEQLLR,FYPELFRQFY,FYPELFRQFYQLDAYPSG,FYPELFRQFYQLDAYPSGAW,PELFRQFYQL,ELFRQFYQLDAYPSGAWYYV,LFRQFYQLDA,RQFYQLDAYP,RQFYQLDAYPSG,RQFYQLDAYPSGAWYYVP,RQFYQLDAYPSGAWYYVPLG,QFYQLDAYPSGAWYYVPL,FYQLDAYPSG,FYQLDAYPSGAWYYVPLGTQYTDAPSFSDI,YQLDAYPSGAWYYVPLGTQY,QLDAYPSGAW,DAYPSGAWYY,DAYPSGAWYYVPLGTQYT,DAYPSGAWYYVPLGTQYTDA,YPSGAWYYVP,YPSGAWYYVPLGTQ,YPSGAWYYVPLGTQYT,PSGAWYYVPLGTQYTDAPSF,SGAWYYVPLG,AWYYVPLGTQ,AWYYVPLGTQYT,AWYYVPLGTQYTDAPSFS,AWYYVPLGTQYTDAPSFSDI,YYVPLGTQYT,YVPLGTQYTDAPSFSDIPNP,VPLGTQYTDA,PLGTQYTDAPSFSDIPNPIGSENSEKTTMPLW,LGTQYTDAPS,LGTQYTDAPSFS,LGTQYTDAPSFSDIPNPI,LGTQYTDAPSFSDIPNPIGS,TQYTDAPSFS,QYTDAPSFSDIP,QYTDAPSFSDIPNPIGSENS,YTDAPSFSDI,YTDAPSFSDIPNPI,YTDAPSFSDIPNPIGSENSEKT,DAPSFSDIPN,DAPSFSDIPNPIGSENSE,DAPSFSDIPNPIGSENSEKT,YVPLGTQYTDAPSFSDIPN,PSFSDIPNPI,SFSDIPNPIGSENSEKTTMP,FSDIPNPIGS,FSDIPNPIGSENSE,DIPNPIGSEN,DIPNPIGSENSEKTTMPL,DIPNPIGSENSEKTTMPLW,PNPIGSENSE,PIGSENSEKT,GSENSEKTTM,GSENSEKTTMPLW,SENSEKTTMPLW,ENSEKTTMPL,SEKTTMPLW,VAVALARPKHPIKHQGLPQEVLNENLLRFFVAPF,AVALARPKHPIKHQGLPQEVLNENLL,PKHPIKHQGLPQEVLNENLLRFFVAPFPEVFGKEKVNELSKDIGSESTEDQAME,KVNELSKDIGSESTEDQAMEDIKQMEAESISSSEEIVPNSVEQKHIQKEDVPSER,QKHIQKEDVPSERYLGYLEQLLRLK,DVPSERYLGYLEQLLRLKKYKVP,YLEQLLRLKKYKVPQLEIVPNSA,LRLKKYKVPQLEIVPNSAEERLHSMKE,QLEIVPNSAEERLHSMKEGIHAQQKEPMIGVNQE,QKEPMIGVNQELAYFYPELFRQFYQLDAYPSGAW,FYQLDAYPSGAWYYVPLGTQYTDA,GAWYYVPLGTQYTDAPSFSDIP
αS2-CN	MKFFIFTCLLAVALAKNTMEHVSSSEESIISQETYKQEKNMAINPSKENLCSTFCKEVVRNANEEEYSIGSSSEESAEVATEEVKITVDDKHYQKALNEINQFYQKFPQYLQYLYQGPIVLNPWDQVKRNAVPITPTLNREQLSTSEENSKKTVDMESTEVFTKKTKLTEEEKNRLNFLKKISQRYQKFALPQYLKTVYQHQKAMKPWIQPKTKVIPYVRYL	222	26.02	KNTMEHVSSS,KNTMEHVSSSEESIISQETY,TMEHVSSSEE,MEHVSSSEESIISQETYKQE,EHVSSSEESI,VSSSEESIIS,VSSSEESIISQETYKQEKNM,SSEESIISQE,SEESIISQETYKQEKNMAIN,EESIISQETY,SIISQETYKQ,SIISQETYKQEKNMAINPSK,ISQETYKQEK,SQETYKQEKNMAINPSKENL,QETYKQEKNM,TYKQEKNMAI,TYKQEKNMAINPSKENLCST,KQEKNMAINP,QEKNMAINPSKENLCSTFCK,EKNMAINPSK,NMAINPSKEN,NMAINPSKENLCSTFCKEVV,AINPSKENLC,INPSKENLCSTFCKEVVRNA,NPSKENLCST,SKENLCSTFC,SKENLCSTFCKEVV,SKENLCSTFCKEVVRNANEE,ENLCSTFCKE,NLCSTFCKEVVRNANEEEYS,LCSTFCKEVV,STFCKEVVRNANEEEYSIGS,TFCKEVVRN,FCKEVVRNAN,CKEVVRNANEEEYSIGSSSE,KEVVRNANEE,VVRNANEEEY,VVRNANEEEYSIGS,VVRNANEEEYSIGSSSEESA,RNANEEEYSI,NANEEEYSIGSSSEESAEVA,ANEEEYSIGS,EEEYSIGSSS,EEEYSIGSSSEESAEVATEE,EYSIGSSSEE,YSIGSSSEESAEVATEEVKI,SIGSSSEESA,GSSSEESAEV,GSSSEESAEVATEEVKITVD,SSEESAEVAT,SEESAEVATEEV,SEESAEVATEEVKITVDDKH,EESAEVATEE,SAEVATEEVK,SAEVATEEVKITVDDKHYQK,EVATEEVKIT,VATEEVKITVDDKHYQKALN,ATEEVKITVD,EEVKITVDDKHYQKALNEIN,EEVKITVDDK,VKITVDDKHY,KITVDDKHYQKALNEINQFY,ITVDDKHYQK,VDDKHYQKALNEINQFYQKF,VDDKHYQKAL,DKHYQKALNE,KHYQKALNEINQFYQKFPQY,HYQKALNEIN,QKALNEINQF,QKALNEINQFYQKFPQYLQY,ALNEINQFYQ,LNEINQFYQKFPQYLQYLYQ,NEINQFYQKF,NEINQFYQKFPQYLQYLY,INQFYQKFPQ,INQFYQKFPQYLQYLYQGPI,QFYQKFPQYL,FYQKFPQYLQYLYQGPIVLN,YQKFPQYLQY,KFPQYLQYLY,KFPQYLQYLYQGPIVLNPWD,PQYLQYLYQG,PQYLQYLYQGPIVL,QYLQYLYQGPIVLNPWDQVK,YLQYLYQGPI,QYLYQGPIVL,QYLYQGPIVLNPWDQVKRNA,LYQGPIVLNP,YQGPIVLNPWDQVKRNAVPI,QGPIVLNPWD,PIVLNPWDQV,PIVLNPWDQVKRNAVPITPT,VLNPWDQVKR,LNPWDQVKRNAVPITPTLNR,NPWDQVKRNA,WDQVKRNAVP,WDQVKRNAVPITPTLNREQL,QVKRNAVPIT,VKRNAVPITPTLNREQLSTS,KRNAVPITPT,NAVPITPTLN,NAVPITPTLNREQLSTSEEN,VPITPTLNRE,VPITPTLNREQL,PITPTLNREQLSTSEENSKK,ITPTLNREQL,PTLNREQLST,PTLNREQLSTSEENSKKTVD,TLNREQLSTSEENSKKTVDM,LNREQLSTSE,NREQLSTSEENSKKTVDMES,REQLSTSEEN,QLSTSEENSK,QLSTSEENSKKTVDMESTEV,STSEENSKKT,TSEENSKKTVDMESTEVFTK,SEENSKKTVD,ENSKKTVDME,ENSKKTVDMESTEVFTKKTK,SKKTVDMEST,KKTVDMESTEVFTKKTKLTE,KTVDMESTEV,VDMESTEVFT,VDMESTEVFTKKTKLTEEEK,MESTEVFTKK,ESTEVFTKKTKLTEEEKNRL,STEVFTKKTK,STEVFTKKTKLTEEEK,EVFTKKTKLT,EVFTKKTKLTEEEKNRLNFL,FTKKTKLTEE,TKKTKLTEEEKNRLNFLKKI,KKTKLTEEEK,TKLTEEEKNR,TKLTEEEKNRLNFLKKISQR,LTEEEKNRLN,TEEEKNRLNFLKKISQRYQK,EEEKNRLNFL,EKNRLNFLKK,EKNRLNFLKKISQRYQ,EKNRLNFLKKISQRYQKFAL,EKNRLNFLKKISQRYQKFALPQYLKT,NRLNFLKKIS,RLNFLKKISQRYQKFALPQY,LNFLKKISQR,FLKKISQRYQ,FLKKISQRYQKFALPQYLKT,KKISQRYQKF,KKISQRYQKFALPQYLKTVYQHQK,KISQRYQKFALPQYLKTVYQ,ISQRYQKFAL,QRYQKFALPQ,QRYQKFALPQYLKTVYQHQK,YQKFALPQYL,QKFALPQYLKTVYQHQKAMK,KFALPQYLKT,ALPQYLKTVY,ALPQYLKTVYQHQKAMKPWI,PQYLKTVYQH,QYLKTVYQHQKAMKPWIQPK,YLKTVYQHQK,KTVYQHQKAM,KTVYQHQKAMKPWIQPKTKV,KTVYQHQKAMKPWIQPKTKVIPYVRYL,VYQHQKAMKP,YQHQKAMKPWIQPKTKVIPY,QHQKAMKPWI,QKAMKPWIQP,QKAMKPWIQPKTKVIPYVRY,AMKPWIQPKT,MKPWIQPKTKVIPYVRYL,KPWIQPKTKV,WIQPKTKVIP,QPKTKVIPYV,KTKVIPYVRY,LAKNTMEHVSSSEESIISQETYKQE,VSSSEESIISQETYKQEKNMAINPS,SQETYKQEKNMAINPSKENLCSTFC,MAINPSKENLCSTFCKEVVRNANEEEYSIGSSSEES,EVVRNANEEEYSIGSSSEESAEVATE,EYSIGSSSEESAEVATEEVKITVDDKH,ESAEVATEEVKITVDDKHYQKALNEI,TEEVKITVDDKHYQKALNEINQFYQKFPQY,YLQYLYQGPIVLNPWDQVKRNAVP,IVLNPWDQVKRNAVPITPTLNREQLSTSEENSKKTVDMESTEVFTKKTKLTEEEKNRLNFLK,EEKNRLNFLKKISQRYQKFALP,KISQRYQKFALPQYLKTVYQHQKAMKPWIQP,HQKAMKPWIQPKTKVIPYVRYL
κ-CN	MMKSFFLVVTILALTLPFLGAQEQNQEQPIRCEKDERFFSDKIAKYIPIQYVLSRYPSYGLNYYQQKPVALINNQFLPYPYYAKPAAVRSPAQILQWQVLSNTVPAKSCQAQPTTMARHPHPHLSFMAIPPKKNQDKTEIPTINTIASGEPTSTPTTEAVE	190	21.27	QEQNQEQPIR,QEQNQEQPIRCEKDERFFSD,QNQEQPIRCE,NQEQPIRCEKDERFFSDKIA,QEQPIRCEKD,QPIRCEKDER,QPIRCEKDERFFSDKIAKYI,IRCEKDERFF,IRCEKDERFFSDKIAKYI,RCEKDERFFSDKIAKYIPIQ,CEKDERFFSD,CEKDERFFSDKIAK,CEKDERFFSDKIAKYI,KDERFFSDKI,KDERFFSDKIAKYIPIQYVL,ERFFSDKIAK,RFFSDKIAKYIPIQYVLSRY,FFSDKIAKYI,SDKIAKYIPI,SDKIAKYIPIQYVLSRYPSY,KIAKYIPIQY,KIAKYIPIQYVLSRYPSYGLNYYQ,IAKYIPIQYVLSRYPSYGLN,AKYIPIQYVL,AKYIPIQYVLSRYP,KYIPIQYVLSRYPSYGLNY,YIPIQYVLSR,YIPIQYVLSRYPSYGLNYYQ,PIQYVLSRYP,IQYVLSRYPSYGLNYYQQKP,QYVLSRYPSY,QYVLSRYPSYGLNY,VLSRYPSYGL,VLSRYPSYGLNYYQQKPVAL,SRYPSYGLNY,RYPSYGLNYYQQKPVALINN,YPSYGLNYYQ,SYGLNYYQQK,SYGLNYYQQKPVALINNQFL,GLNYYQQKPV,LNYYQQKPVALINNQFLPYP,NYYQQKPVAL,YQQKPVALIN,YQQKPVALINNQFLPYPYYA,QKPVALINNQ,KPVALINNQFLPYPYYAKPA,PVALINNQFL,PVALINNQFLPYPYYAKPAAVR,ALINNQFLPY,ALINNQFLPYPYYAKPAAVR,INNQFLPYPY,NNQFLPYPYYAKPAAVRSPA,NQFLPYPYYA,NQFLPYPYYAKP,NQFLPYPYYAKPAA,FLPYPY

	STVATLEDSPEVIESPPEINTVQVTSTAV			YAKP,FLPYPYYAKPAAVRSPAQIL,PYPYYAKPAA,YPYYAKPAAVRSPAQILQWQ,PYYAKPAAVR,YAKPAAVRSPAQILQWQVLS,YAKPAAVRSP,KPAAVRSPAQ,PAAVRSPAQILQWQVLSNTV,AAVRSPAQIL,VRSPAQILQWQVLSNTVPAK,VRSPAQILQW,VRSPAQILQWQV,RSPAQILQWQVL,SPAQILQWQV,PAQILQWQVLSNTVPAKSCQ,AQILQWQVLS,ILQWQVLSNT,ILQWQVLSNTVPAKSCQAQP,QWQVLSNTVP,WQVLSNTVPAKSCQAQPTTM,QVLSNTVPAK,LSNTVPAKSC,NTVPAKSCQA,TVPAKSCQAQPTTMARHPHP,VPAKSCQAQP,AKSCQAQPTT,AKSCQAQPTTMARHPHPHLS,SCQAQPTTMA,CQAQPTTMARHPHPHLSFMA,QAQPTTMARH,QPTTMARHPH,QPTTMARHPHPHLSFMAIPP,TTMARHPHPH,TMARHPHPHLSFMAIPPKKN,MARHPHPHLS,MARHPHPHLSFMAIPPKKNQDK,RHPHPHLSFM,RHPHPHLSFMAI,RHPHPHLSFMAIPPKKNQ,RHPHPHLSFMAIPPKKNQDK,PHPHLSFMAI,HPHLSFMAIPPKKNQDKTEI,PHLSFMAIPP,PHLSFMAIPPKKNQ,LSFMAIPPKK,LSFMAIPPKKNQDKTEIPTI,FMAIPPKKNQ,MAIPPKKNQDKTEIPTINTI,AIPPKKNQDK,PPKKNQDKTE,PPKKNQDKTEIPTINTIASG,KKNQDKTEIP,KKNQDKTEIPTINT,KKNQDKTEIPTINTIA,KNQDKTEIPTINTIASGEPT,NQDKTEIPTI,DKTEIPTINT,DKTEIPTINTIASGEPTSTP,TEIPTINTIA,EIPTINTIASGEPTSTPTTE,IPTINTIASG,TINTIASGEP,TINTIASGEPTSTPTTEAVE,NTIASGEPTS,TIASGEPTSTPTTEAVESTV,IASGEPTSTP,SGEPTSTPTT,SGEPTSTPTTEAVESTVATL,EPTSTPTTEA,PTSTPTTEAVESTVATLEDS,TSTPTTEAVE,TPTTEAVEST,TPTTEAVESTVATLEDSPEV,TTEAVESTVA,TEAVESTVATLEDSPEVIES,EAVESTVATL,EAVESTVATLED,AVESTVATLE,VESTVATLED,VESTVATLEDSPEVIESPPE,STVATLEDSP,TVATLEDSPEVIESPPEINT,VATLEDSPEV,TLEDSPEVIE,TLEDSPEVIESPPEINTVQV,EDSPEVIESP,DSPEVIESPPEINTVQVTST,SPEVIESPPE,SPEVIESPPEINTVQVTS,EVIESPPEIN,EVIESPPEINTVQVTSTAV,IESPPEINTV,IESPPEINTVQVTS,SPPEINTVQV,ESPPEINTVQVTS,PEINTVQVTS,INTVQVTSTA,GAQEQNQEQPIRCEKDERFFSD,QEQPIRCEKDERFFSDKIAKYIPIQY,KDERFFSDKIAKYIPIQYVLSRY,RFFSDKIAKYIPIQYVLSRYPSY,AAVRSPAQILQWQVLSNTVPAKSCQAQPTTMARHPH,TTEAVESTVATLEDSPEVIESP,VESTVATLEDSPEVIESPPEIN,STVATLEDSPEVIESPPEINTVQVTSTAV
βLG	MKCLLLALALTCGAQALIVTQTMKGLDIQKVAGTWYSLAMAASDISLLDAQSAPLRVYVEELKPTPEGDLEILLQKWENDECAQKKIIAEKTKIPAVFKIDALNENKVLVLDTDYKKYLLFCMENSAEPEQSLACQCLVRTPEVDDEALEKFDKALKALPMHIRLSFNPTQLEEQCHI	178	19.94	LIVTQT,LIVTQTMK,LIVTQTMKGL,LIVTQTMKGLDIQK,LIVTQTMKGLDIQKVA,LIVTQTMKGLDIQKVAGTWY,LIVTQTMKGLDIQKVAGTWYS,IVTQTMKGLD,VTQTMK,VTQTMKGLDI,TQTMKGLDIQ,TQTMKGLDIQKVAGTWYSLA,QTMKGL,QTMKGLDIQK,TMKGLDIQKV,MKGLDI,MKGLDIQKVA,MKGLDIQKVAGTWYSLAMAA,KGLDIQKVAG,KGLDIQKVAGTWYSLAM,GLDIQK,GLDIQKVAGT,LDIQKVAGTW,LDIQKVAGTWYSLAMAASDI,DIQKVA,DIQKVAGTWY,DIQKVAGTWYSLAM,IQKVAGTWYS,QKVAGT,QKVAGTWYSL,QKVAGTWYSLAMAAS,QKVAGTWYSLAMAASDISLL,KVAGTWYSLAMAASDI,VAGTWYSLAM,VAGTWYSLAMAA,AGTWYSLAMAASDISLLDAQ,GTWYSLAMAA,WYSLAMAASD,WYSLAMAASDIS,WYSLAMAASDISLLDAQSAP,YSLAMAASDI,YSLAMAASDISL,SLAMAASDIS,SLAMAASDISLL,LAMAASDISL,LAMAASDISLLD,LAMAASDISLLDAQSAPLRV,AMAASDISLL,AMAASDISLLDA,MAASDISLLD,MAASDISLLDAQSAPLRV,AASDIS,AASDISLLDA,AASDISLLDAQSAPLR,AASDISLLDAQSAPLRVYVE,ASDISLLDAQ,SDISLL,SDISLLDAQS,DISLLDAQSA,DISLLDAQSAPLRVYVEELK,ISLLDA,ISLLDAQSAP,SLLDAQSAPL,SLLDAQSAPLRVYVEELK,LLDAQS,LLDAQSAPLR,LLDAQSAPLRVYVEELKP,LLDAQSAPLRVYVEELKPTP,LDAQSAPLRV,DAQSAP,DAQSAPLRVY,AQSAPLRVYVEELKPTPEGD,QSAPLR,QSAPLRVYVE,QSAPLRVYVEEL,SAPLRVYVEE,APLRVY,APLRVYVEEL,APLRVYVEELKPTPEGDLEI,APLRVYVEELKPTPEGDLEIL,PLRVYVEELK,LRVYVEELKP,RVYVEELKPT,RVYVEELKPTPEGDLEILLQ,VYVEELKPTP,VYVEELKPTPEGDLEILLQK,YVEELKPTPE,VEELKPTPEG,VEELKPTPEGDLEILLQKWE,EELKPTPEGD,ELKPTPEGDL,LKPTPEGDLE,LKPTPEGDLEILLQKWENGE,KPTPEGDLEI,KPTPEGDLEILLQK,KPTPEGDLEILLQKWENDECA,PTPEGDLEIL,TPEGDL,TPEGDLEILL,TPEGDLEILLQKWENGECAQ,PEGDLEILLQ,EGDLEI,EGDLEILLQK,GDLEILLQKW,GDLEILLQKWENGECAQKKI,DLEILL,DLEILLQKWE,LEILLQKWEN,EILLQK,EILLQKWEND,ILLQKWENDE,LLQKWE,LLQKWENDEC,LLQKWENGEC,LLQKWENDECAQKKIIAEKTK,LQKWENDECA,LQKWENDECAQKKIIAEKTK,QKWEND,QKWENDECAQ,QKWENGECAQ,KWENDECAQK,WENDEC,WENDECAQKK,ENDECAQKKI,NDECAQ,NDECAQKKII,DECAQKKIIA,ECAQKK,ECAQKKIIAE,CAQKKIIAEK,AQKKII,AQKKIIAEKT,AQKKIIAEKTKI,AQKKIIAEKTKIPAVFKIDA,AQKKIIAEKTKIPAVFKIDAL,QKKIIAEKTK,KKIIAE,KKIIAEKTKI,KIIAEKTKIP,KIIAEKTKIPAVFKIDALNE,IIAEKT,IIAEKTKIPA,IAEKTKIPAV,AEKTKI,AEKTKIPAVF,AEKTKIPAVFKIDALNENKV,EKTKIPAVFK,KTKIPA,KTKIPAVFKI,KTKIPAVFKIDA,TKIPAVFKID,TKIPAVFKIDALNENKVLVL,KIPAVF,KIPAVFKIDA,KIPAVFKIDALNENKVLVLDT,IPAVFKIDAL,PAVFK,PAVFKI,PAVFKIDALN,PAVFKIDALNENKVLVLDTD,AVFKIDALNE,VFKIDA,VFKIDALNEN,FKIDALNENK,FKIDALNENKVLVLDTDYKK,KIDALN,KIDALNENKV,IDALNENK,IDALNENKVL,DALNEN,DALNENKVLV,DALNENKVLVLD,DALNENKVLVLDTDYKKYLL,ALNENKVLVL,LNENKV,LNENKVLVLD,LNENKVLVLDTDYKKYLLFCM,NENKVLVLDT,NENKVLVLDTDYKKYLLFCM,ENKVLV,ENKVLVLDTD,NKVLVLDTDY,KVLVLD,KVLVLDTDYK,KVLVLDTDYKKYLLFCMENS,VLVLDTDYK,VLVLDTDYKK,LVLDTD,LVLDTDYKKY,VLDTDYKKYL,VLDTDYKKYLLFCMENSAEP,LDTDYK,LDTDYKKYLL,TDYKKY,TDYKKYLLFC,TDYKKYLLFCME,TDYKKYLLFCMENSAEPEQS,TDYKKYLLFCMENSAEPEQSL,YKKYLL,YKKYLLFCME,KKYLLFCMENSAEPEQSLAC,KYLLFC,KYLLFCMENS,YLLFCMENSAE,YLLFCMENSAEPEQSLVCQCLVR,LLFCME,LLFCMENSAE,LLFCMENSAEPEQSLACQCL,FCMENS,FCMENSAEPE,CMENSAEPEQ,CMENSAEPEQSLACQCLVRT,MENSAE,MENSAEPEQS,ENSAEPEQSL,ENSAEPEQSLVCQCLVRTPEVDDEALEKFDKALKALPM,NSAEPE,NSAEPEQSLA,NSAEPEQSLACQCLVRTPEV,SAEPEQSLAC,AEPEQS,AEPEQSLACQ,EPEQSLACQC,EPEQSLACQCLVRTPEVDDE,PEQSLA,PEQSLACQCL,EQSLACQCLV,QSLACQ,QSLACQCLVR,QSLACQCLVRTPEVDDEALE,SLACQCLVRT,LACQCLVRTP,LACQCLVRTPEV,ACQCLVRTPE,ACQCLVRTPEVDDEALEKFD,CQCLVRTPEV,QCLVRTPEVD,CLVRTPEVDD,CLVRTPEVDDEALEKFDKAL,LVRTPEVDDE,VRTPEVDDEA,RTPEVDDEAL,RTPEVDDEALE,RTPEVDDEALEKFDKALKAL,TPEVDD,TPEVDDEALE,TPEVDDEALEK,PEVDDEALEK,EVDDEA,EVDDEALEKF,EVDDEALEKFDKALKALP,EVDDEALEKFDKAL,EVDDEALEKFDKALKALPMH,VDDEALEKFD,DDEALE,DDEALEKFDK,DEALEKFDKA,DEALEKFDKALKALPMHIRL,EALEKF,EALEKFDKAL,ALEKFDKALK,ALEKFDKALKALPMHIRLSFN,LEKFDK,LEKFDKALKA,LEKFDKALKALPMHIRLSFN,EKFDKALKAL,KFDKAL,KFDKALKALP,FDKALKALPM,FDKALKALPMHIRLSFNPTQ,DKALKA,DKALKALPMH,KALKALPMHI,ALKALP,ALKALPMHIR,ALKALPMHIRLSFNPTQLEE,LKALPMHIRL,KALPMH,KALPMHIRLS,KALPMHIRLSFN,ALPMHIRLSF,ALPMHIRLSFNPTQLEEQCH,ALPMHIRLSFNPTQLEEQCHI,LPMHIR,LPMHIRLSFN,PMHIRLSFNP,MHIRLS,MHIRLSFNPT,MHIRLSFNPTQLEEQCHI,HIRLSFNPTQLEEQCHI,HIRLSFNPTQ,IRLSFN,IRLSFNPTQL,RLSFNPTQLE,LSFNPT,LSFNPTQLEE,LSFNPTQLEEQCHI,SFNPTQLEEQ,FNPTQLEEQC,FNPTQLEEQCHI,NPTQLEEQCH,PTQLEEQCHI,MKCLLLALALTCGAQALIVTQTMKGLDIQKVAGT,AQKKIIAEKTKIPAVFKIDALNENKVLVLDTDYK,LFCMENSAEPEQSLACQCLVRTP,ALEKFDKALKALPMHIRLSFNPTQLEEQCHI

αLA	MMSFVSLLLVGILFHATQAEQLTKCEVFRELKDLKGYGGVSLPEWVCTTFHTSGYDTQAIVQNNDSTEYGLFQINNKIWCKDDQNPHSSNICNISCDKFLDDDLTDDIMCVKKILDKVGINYWLAHKALCSEKLDQWLCEKL	142	16.25	EQLTKCEVFR,EQLTKCEVFRELKDLK,LTKCEVFREL,KCEVFRELKD,KCEVFRELKDLKGY,EVFRELKDLK,FRELKDLKGY,ELKDLKGYGG,KDLKGYGGVS,KDLKGYGGVSLPEW,LKGYGGVSLP,GYGGVSLPEW,GYGGVSLPEWVCTTFHTSGYDTQAIVQNNDSTEYGLFQINNK,GGVSLPEWVC,VSLPEWVCTT,LPEWVCTTFH,EWVCTTFHTS,VCTTFHTSGY,TTFHTSGYDT,FHTSGYDTQA,TSGYDTQAIV,GYDTQAIVQN,DTQAIVQNND,QAIVQNNDST,IVQNNDSTEY,QNNDSTEYGL,NDSTEYGLFQ,STEYGLFQIN,STEYGLFQINNK,EYGLFQINNK,GLFQINNKIW,FQINNKIWCK,INNKIWCKDD,NKIWCKDDQN,IWCKDDQNPHSSNICNISCDKFLDDDLTDDIMCVK,IWCKDDQNPHSSNICNISCDKFLDDDLTDDIMCVKK,IWCKDDQNPH,CKDDQNPHSS,QNPHSSNICN,PHSSNICNIS,SSNICNISCD,NICNISCDKF,CNISCDKFLD,ISCDKFLDDD,CDKFLDDDLT,KFLDDDLTDD,LDDDLTDDIM,DDLTDDIMCV,LTDDIMCVKK,DDIMCVKKIL,IMCVKKILDK,CVKKILDKVG,KKILDKVGIN,ILDKVGINYW,DKVGINYWLA,VGINYWLAHK,INYWLAHKAL,YWLAHKALCS,LAHKALCSEK,HKALCSEKLD,ALCSEKLDQW,ALCSEKLDQWLCEKL,CSEKLDQWLC,EKLDQWLCEK,DDQNPHSSNI,MMSFVSLLLVGILFHATQAEQL
BSA	MKWVTFISLLLLFSSAYSRGVFRRDTHKSEIAHRFKDLGEEHFKGLVLIAFSQYLQQCPFDEHVKLVNELTEFAKTCVADESHAGCEKSLHTLFGDELCKVASLRETYGDMADCCEKQEPERNECFLSHKDDSPDLPKLKPDPNTLCDEFKADEKKFWGKYLYEIARRHPYFYAPELLYYANKYNGVFQECCQAEDKGACLLPKIETMREKVLASSARQRLRCASIQKFGERALKAWSVARLSQKFPKAEFVEVTKLVTDLTKVHKECCHGDLLECADDRADLAKYICDNQDTISSKLKECCDKPLLEKSHCIAEVEKDAIPENLPPLTADFAEDKDVCKNYQEAKDAFLGSFLYEYSRRHPEYAVSVLLRLAKEYEATLEECCAKDDPHACYSTVFDKLKHLVDEPQNLIKQNCDQFEKLGEYGFQNALIVRYTRKVPQVSTPTLVEVSRSLGKVGTRCCTKPESERMPCTEDYLSLILNRLCVLHEKTPVSEKVTKCCTESLVNRRPCFSALTPDETYVPKAFDEKLFTFHADICTLPDTEKQIKKQTALVELLKHKPKATEEQLKTVMENFVAFVDKCCAADDKEACFAVEGPKLVVSTQTALA	607	69.3	ESHAGCEKS,DDSPDLPKLKPDPNTLC,LPKLKPDPNTLCDEFKAD,CDEFKADEKKFWGKY,EFKAD,EFKADE,EFKADEKK,FKADEK,FKADEKKFWGKYL,FKADEKKFWGKYLYE,FKADEKKFWGKYLYEIAR,KADEKK,ADEKK,ADEKKFWGKYLYEIAR,YANKYNGV,EDKGACLLPKIE,EKDAIPENLPPLTADFAEDK,HPEYAVSVLL,EYAV,PHACYSTVFDKLKHLVDEP,NCDQFEKLG,VGTRCCTKPESERM,LSLILNRLC,LILNR,PKAFDEKLFT,TLPDTEKQI,VMENFVAF,ENFV,LVVSTQTAL,LPKLKPDPNTLCDEFKADEKKFWGKYLY,KLKPDPNTLCDEFKADEKKFWGK

Note. β-CN: β-casein; αS1-CN: αS1-casein; αS2-CN: αS2-casein; κ-CN: κ-casein; βLG: β-lactoglobulin; αLA: α-lactalbumin; BSA: bovine serum albumin.

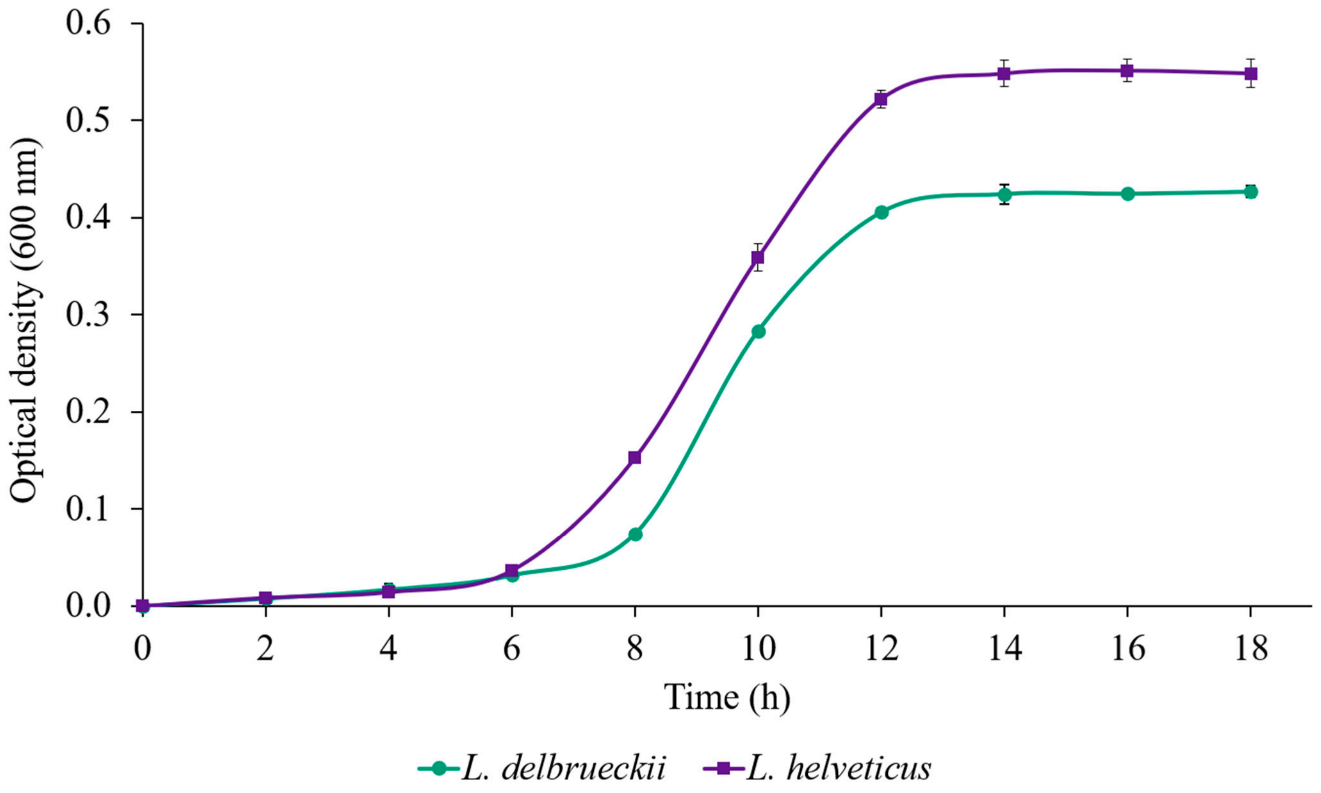

Figure S1: Growth profile of *L. delbrueckii* and *L. helveticus* strains, determined by optical density (OD_{600}), in MRS broth inoculated with 1% (v/v) of the bacterial culture. Data are presented as mean ± standard deviation of triplicate measurements.

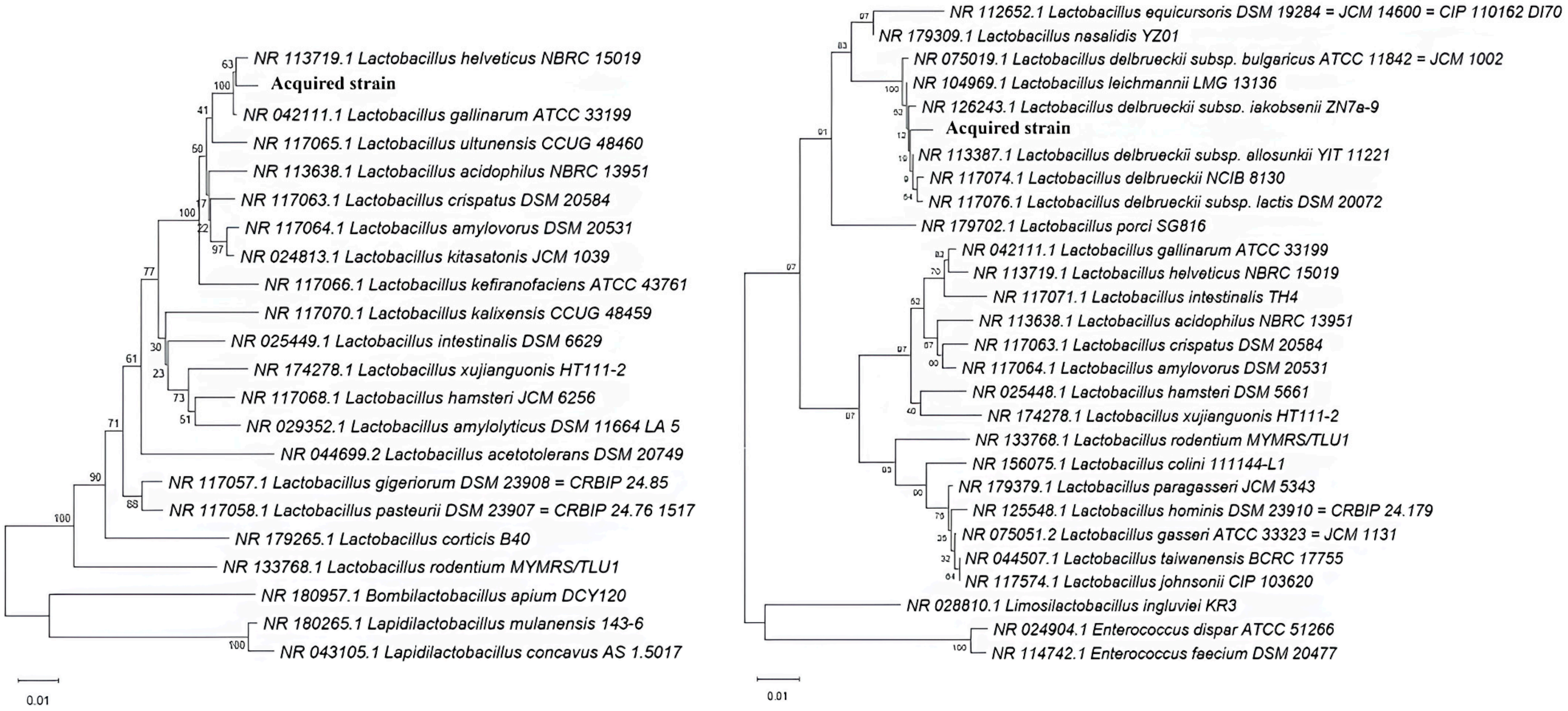

Figure S2: Phenetic tree showing the phylogenetic distance between the acquired bacterial strains based on 16S rRNA. Constructed using MEGA 11 software via the Neighbor-Joining method with the Jukes-Cantor parameter and 1,000 bootstrap replicates. The identification was performed using one sample of each bacterial strain.

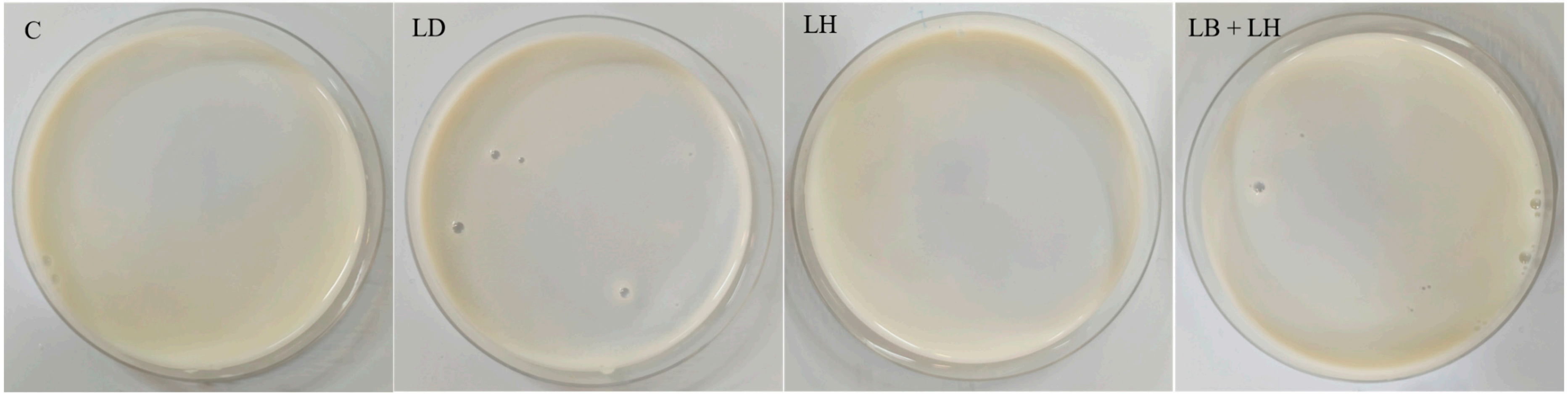

Figure S3: Visual appearance of reconstituted milk and fermented milk samples. C: reconstituted milk (control); LD: fermented milk with *L. delbrueckii*; LH: fermented milk with *L. helveticus*; LD + LH: fermented milk with *L. delbrueckii* and *L. helveticus*. Images were obtained from pooled samples prepared from triplicate biological replicates.

Interestingly, the protein showing the highest theoretical susceptibility to hydrolysis was not the one with the greatest reduction in immunoreactivity observed experimentally, reinforcing that the extent of hydrolysis and epitope destruction are not necessarily directly related. This observation indicates that the biological potential of the released peptides depends not only on the extent of hydrolysis but also on the amino acid sequence and the location of the enzymatic cleavage sites. All proteins generated peptides with predicted ACE-, DPP-IV-inhibitory, and antioxidant activities (**Table S2**).

Table S2: Simulated enzymatic hydrolysis of bovine milk proteins by the enzyme lactocepin (EC 3.4.21.96) using the BIOPEP-UWM platform.

Protein	Results of enzyme action	Bioactivities
β-CN	MKV - L - I - L - AC - L - V - A - L - A - L - ARE - L - EE - L - NV - P - GEIV - E - S - L - S - S - SEE - SITRINKKIEKF - Q - SEE - Q - Q - Q - TEDE - L - Q - DKIH - P - FA - Q - T - Q - S - L - V - Y - P - F - P - G - P - I - P - N - S - L - P - Q - NI - P - P - L - T - Q - T - P - V - V - V - P - P - F - L - Q - P - EV - MGV - SKV - KEAMA - P - KHKEM - P - F - P - KY - P - V - E - P - FTE - S - Q - S - L - T - L - TDV - EN - L - H - L - P - L - P - L - L - Q - SWMH - Q - P - H - Q - P - L - P - P - TV - MF - P - P - Q - SV - L - S - L - S - Q - SKV - L - P - V - P - Q - KAV - P - Y - P - Q - RDM - P - I - Q - AF - L - L - Y - Q - E - P - V - L - G - P - V - RG - P - F - P - IIV	ACE inhibitor<br>Alanine carboxypeptidase inhibitor<br>Antioxidative<br>Citrate lyase deacetylase inhibitor<br>Dipeptidyl peptidase III inhibitor<br>Dipeptidyl peptidase IV inhibitor<br>Glutamate carboxypeptidase inhibitor<br>Glutamate carboxypeptidase II inhibitor<br>Hypotensive<br>Inhibitor of tripeptidyl peptidase II<br>Leucyltransferase inhibitor<br>Neprilysin inhibitor<br>Stimulating<br>Thymidylate synthase inhibitor
αS1-CN	MK - L - L - I - L - TC - L - V - AV - A - L - AR - P - KH - P - IKH - Q - G - L - P - Q - EV - L - NEN - L - L - RFFV - A - P - F - P - EV - FGKEKV - NE - L - SKDIG - SE - STED - Q - AMEDIK - Q - MEAE - SI - S - S - SEEIV - P - N - SV - E - Q - KHI - Q - KEDV - P - SERY - L - GY - L - E - Q - L - L - R - L - KKYKV - P - Q - L - EIV - P - N - SAEER - L - H - SMKEGIHA - Q - Q - KE - P - MIGV - N - Q - E - L - AYFY - P - E - L - FR - Q - FY - Q - L - DAY - P - SGAWYYV - P - L - GT - Q - YTDA - P - SF - SDI - P - N - P - IG - SEN - SEKTTM - P - L - W	ACE inhibitor<br>Alpha-amylase inhibitor<br>Alpha-glucosidase inhibitor<br>Antioxidative<br>Dipeptidyl peptidase III inhibitor<br>Dipeptidyl peptidase IV inhibitor<br>Hypotensive

		Hypouricemic<br>Inhibitor of tripeptidyl peptidase II<br>Neprilysin inhibitor<br>Renin inhibitor<br>Stimulating
αS2-CN	MKFFIFTC - L - L - AV - A - L - AKNTMEHV - S - S - SEE - SII - S - Q - ETYK - Q - EKNMAIN - P - SKEN - L - C - STFCKEV - V - RNANEEEY - SIG - S - S - SEE - SAEV - ATEEV - KITV - DDKHY - Q - KA - L - NEIN - Q - FY - Q - KF - P - Q - Y - L - Q - Y - L - Y - Q - G - P - IV - L - N - P - WD - Q - V - KRNAV - P - IT - P - T - L - NRE - Q - L - ST - SEEN - SKKTV - DME - STEV - FTKKTK - L - TEEEKNR - L - NF - L - KKI - S - Q - RY - Q - KFA - L - P - Q - Y - L - KTV - Y - Q - H - Q - KAMK - P - WI - Q - P - KTKV - I - P - YV - RY - L	ACE inhibitor<br>Alpha-amylase inhibitor<br>Alpha-glucosidase inhibitor<br>Antioxidative<br>CaMPDE inhibitor<br>Dipeptidyl peptidase III inhibitor<br>Dipeptidyl peptidase IV inhibitor<br>Hypotensive<br>PPARγ antagonist<br>Renin inhibitor<br>Stimulating
κ-CN	MMK - SFF - L - V - V - TI - L - A - L - T - L - P - F - L - GA - Q - E - Q - N - Q - E - Q - P - IRCEKDERFF - SDKIAKYI - P - I - Q - YV - L - SRY - P - SYG - L - NYY - Q - Q - K - P - V - A - L - INN - Q - F - L - P - Y - P - YYAK - P - AAV - R - S - P - A - Q - I - L - Q - W - Q - V - L - SNTV - P - AK - SC - Q - A - Q - P - TTMARH - P - H - P - H - L - SFMAI - P - P - KKN - Q - DKTEI - P - TINTIA - SGE - P - T - ST - P - TTEAV - E - STV - AT - L - ED - S - P - EV - IE - S - P - P - EINTV - Q - V - T - STAV	ACE inhibitor<br>Alanine carboxypeptidase inhibitor<br>Antioxidative<br>Dipeptidyl peptidase IV inhibitor<br>Glutamate carboxypeptidase inhibitor<br>Glutamate carboxypeptidase II inhibitor<br>Neprilysin inhibitor
βLG	MKC - L - L - L - A - L - A - L - TCGA - Q - A - L - IV - T - Q - TMKG - L - DI - Q - KV - AGTWY - S - L - AMAA - SDI - S - L - L - DA - Q - SA - P - L - RV - YV - EE - L - K - P - T - P - EGD - L - EI - L - L - Q - KWENDECA - Q - KKIIAEKTKI - P - AV - FKIDA - L - NENKV - L - V - L - DTDYKKY - L - L - FCMEN - SAE - P - E - Q - S - L - AC - Q - C - L - V - RT - P - EV - DDEA - L - EKFDKA - L - KA - L - P - MHIR - L - SFN - P - T - Q - L - EE - Q - CHI	ACE inhibitor<br>Antioxidative<br>Binding<br>Citrate lyase deacetylase inhibitor<br>Dipeptidyl peptidase III inhibitor<br>Dipeptidyl peptidase IV inhibitor<br>Glutamate carboxypeptidase inhibitor<br>Glutamate carboxypeptidase II inhibitor

αLA	MM - SFV - S - L - L - L - V - GI - L - FHAT - Q - AE - Q - L - TKCEV - FRE - L - KD - L - KGYGGV - S - L - P - EWV - CTTFHT - SGYDT - Q - AIV - Q - NND - STEYG - L - F - Q - INNKIWCKDD - Q - N - P - H - S - SNICNI - SCDKF - L - DDD - L - TDDIMCV - KKI - L - DKV - GINYW - L - AHKA - L - C - SEK - L - D - Q - W - L - CEK - L	ACE inhibitor<br>Alanine carboxypeptidase inhibitor<br>Alpha-glucosidase inhibitor<br>Antioxidative<br>CaMPDE inhibitor<br>Dipeptidyl peptidase III inhibitor<br>Dipeptidyl peptidase IV inhibitor<br>Hypolipidemic<br>Hypotensive<br>Neprilysin inhibitor<br>Renin inhibitor<br>Stimulating<br>Tubulin-tyrosine ligase inhibitor
BSA	MKWV - TFI - S - L - L - L - L - F - S - SAY - SRGV - FRRDTHK - SEIAHRFKD - L - GEEHFKG - L - V - L - IAF - S - Q - Y - L - Q - Q - C - P - FDEHV - K - L - V - NE - L - TEFAKTCV - ADE - SHAGCEK - S - L - HT - L - FGDE - L - CKV - A - S - L - RETYGDMADCCEK - Q - E - P - ERNECF - L - SHKDD - S - P - D - L - P - K - L - K - P - D - P - NT - L - CDEFKADEKKFWGKY - L - YEIARRH - P - YFYA - P - E - L - L - YYANKYNGV - F - Q - ECC - Q - AEDKGAC - L - L - P - KIETMREKV - L - A - S - SAR - Q - R - L - RCA - SI - Q - KFGERA - L - KAW - SV - AR - L - S - Q - KF - P - KAEFV - EV - TK - L - V - TD - L - TKV - HKECCHGD - L - L - ECADDRAD - L - AKYICDN - Q - DTI - S - SK - L - KECCDK - P - L - L - EK - SHCIAEV - EKDAI - P - EN - L - P - P - L - TADFAEDKDV - CKNY - Q - EAKDAF - L - G - SF - L - YEY - SRRH - P - EYAV - SV - L - L - R - L - AKEYEAT - L - EECCAKDD - P - HACY - STV - FDK - L - KH - L - V - DE - P - Q - N - L - IK - Q - NCD - Q - FEK - L - GEYGF - Q - NA - L - IV - RYTRKV - P - Q - V - ST - P - T - L - V - EV - SR - S - L - GKV - GTRCCTK - P - E - SERM - P - CTEDY - L - S - L - I - L - NR - L - CV - L - HEKT - P - V - SEKV - TKCCTE - S - L - V - NRR - P - CF - SA - L - T - P - DETYV - P - KAFDEK - L - FTFHADICT - L - P - DTEK - Q - IKK - Q - TA - L - V - E - L - L - KHK - P - KATEE - Q - L - KTV - MENFV - AFV - DKCCAADDKEACFAV - EG - P - K - L - V - V - ST - Q - TA - L - A	ACE inhibitor<br>Antioxidative<br>CaMPDE inhibitor<br>Dipeptidyl peptidase IV inhibitor<br>Glutamate carboxypeptidase II inhibitor<br>HMG-CoA reductase inhibitor<br>Neprilysin inhibitor<br>Neuropeptide<br>Renin inhibitor<br>Stimulating

Note: β-CN: β-casein; αS1-CN: αS1-casein; αS2-CN: αS2-casein; κ-CN: κ-casein; βLG: β-lactoglobulin; αLA: α-lactalbumin; BSA: bovine serum albumin; ACE: angiotensin-converting enzyme; DPP: dipeptidyl peptidase; TPP: tripeptidyl peptidase; CaMPDE: calmodulin-sensitive cyclic nucleotide phosphodiesterase; PPARγ: peroxisome proliferator-activated receptor gamma; HMG-CoA: 3-hydroxy-3-methylglutaryl-coenzyme A.
